# Genome-wide association mapping of sodium and potassium concentration in rice grains and shoots under alternate wetting and drying and continuously flooded irrigation

**DOI:** 10.1007/s00122-021-03828-9

**Published:** 2021-05-04

**Authors:** Caijin Chen, Anthony J. Travis, Mahmud Hossain, Md Rafiqul Islam, Adam H. Price, Gareth J. Norton

**Affiliations:** 1 School of Biological Sciences, University of Aberdeen, Aberdeen, AB24 3UU UK; 2 Department of Soil Science, Bangladesh Agricultural University, Mymensingh, Bangladesh

## Abstract

**Key message:**

**Identification of a large number of QTL and candidate genes for sodium accumulation in a field grown population of rice derived from the** ***aus*** **subpopulation.**

**Abstract:**

Rice (*Oryza sativa* L.) is a globally important cereal crop. Sodium (Na^+^) and potassium (K^+^) are the major monovalent ions which affect rice growth, and exploring their uptake mechanisms will be useful for understanding rice biology. Since the balance of Na^+^ and K^+^ plays a significant role in adaptation of rice to salinity, that biology might inform the search for tolerance. In this study, the Na^+^ and K^+^ concentration and Na^+^/K^+^ ratio in grains and shoots were analyzed in the Bengal and Assam Aus Panel grown in field conditions under continuously flooded (CF) and alternate wetting and drying (AWD) irrigation. Overall, AWD irrigation significantly reduced the Na^+^ concentration and increased the K^+^ concentration in shoots and grains compared to the plants grown under CF. Genome-wide association mapping was conducted on Na^+^, K^+^ concentration and Na^+^/K^+^ ratio with 2 million SNPs using an efficient mixed model. Only QTLs which contained more than two significant SNPs (*p* < 0.0001) and where at least one of these significant SNPs passed a 10% false discovery rate were reported. A total of 106 QTLs were identified as being associated with Na^+^ concentration and Na^+^/K^+^ ratio across all traits and field conditions, with 48 QTLs found in multiple traits and/or water conditions. Four notable QTLs (one each on chromosomes 1 and 11, two on chromosome 2) and the haplotype variants of four candidate genes (*OsHKT1;5*, *OsNHX2*, *LOC_Os02g32490* and *OsFAD2_1*) are discussed. The QTLs/candidate genes identified here could be useful for breeding rice that accumulates lower concentrations of sodium.

**Supplementary Information:**

The online version contains supplementary material available at 10.1007/s00122-021-03828-9.

## Introduction

Rice (*Oryza sativa* L.) is the most important staple food crop in many countries. It is the main food source of over half of the world population, accounting for about 50–80% of daily calorie intake (Solis et al. 2020). The demand for rice is rising due to increasing populations in countries where rice is the staple food and the growing global popularity of rice cuisines (Solis et al. 2020). Na^+^ and K^+^ are the major monovalent ions affecting rice growth, with K^+^ a major macronutrient for plant growth. In addition, the plant’s ability to maintain Na^+^ and K^+^ balance is the key feature of tolerance under salt stress in rice (Negrão et al. 2011). Salt stress is a major constraint to rice acreage and production worldwide (Rohila et al. 2019). It is reported that approximately 960 million hectares of soil is salt impacted globally (Hu et al. 2012). The typical mechanisms of salinity tolerance in rice are Na^+^ exclusion or reduced uptake, and increased absorption of K^+^ to maintain a suitable Na^+^–K^+^ balance in shoots. (Negrão et al. 2011). Thus, understanding the accumulation of Na^+^ and K^+^ in rice is important for exploring the fundementals of rice biology and should provide useful information relevant to rice adaptation to salinity stress.

In recent decades, many quantitative trait loci (QTL) associated with Na^+^ and K^+^ concentration have been identified in rice using biparental mapping populations (Koyama et al. 2001; Bonilla et al. 2002; Lin et al. 2004; Ming-Zhe et al. 2005; Sabouri and Sabouri 2008; Ammar et al. 2009; Pandit et al. 2010; Islam et al. 2011; Ghomi et al. 2013; Hossain et al. 2015). For example, *Saltol*, a major QTL for Na^+^ uptake and Na^+^/K^+^ ratio was mapped to chromosome 1 from recombinant inbred lines derived from a cross between Pokkali (salt tolerant) and IR 29 (salt sensitive) cultivars (Bonilla et al. 2002). The *OsHKT1;5* gene under the *Saltol* locus regulating K^+^/Na^+^ homeostasis in the salt-tolerant *indica* rice cultivar Nona Bokra has been cloned using a map-based approach (Ren et al. 2005). Two major QTLs with very large effects, *qSNC-7* for shoot Na^+^ concentration and *qSKC-1* for shoot K^+^ concentration, have been identified in rice (Lin et al. 2004). There are several genome-wide association (GWA) studies in rice mapping Na^+^, K^+^ concentration and/or Na^+^/K^+^ ratio (Kumar et al. 2015; Batayeva et al. 2018; Frouin et al. 2018; Patishtan et al. 2018; Yang et al. 2018). For instance, a total of 20 loci associated with Na^+^/K^+^ ratio were identified, using 6000 SNPs from 220 rice accessions (Kumar et al. 2015). Eighteen QTLs were associated with Na^+^, K^+^ concentration, and Na^+^/K^+^ ratio, using 68,786 SNPs from 191 temperate *japonica* rice accessions (Batayeva et al. 2018). A significant QTL on chromosome 1–11.48 Mb (same position with *Saltol*) associated with Na^+^ accumulation was identified, using 6.4 million SNPs from 529 rice cultivars in different field trials without salt stress imposed; *OsHKT1;5* was reported to be the gene responsible for this QTL (Yang et al. 2018).

Rice requires large quantities of fresh water to maintain high yields, especially in the dry season (Norton et al. 2018). It is estimated that an average of 2500 L of water is needed to produce 1 kg of rice (Bouman 2009). To reduce the volume of water needed for irrigation during the dry season, the method of alternate wetting and drying (AWD) is being promoted in some countries in Asia (Bouman and Tuong 2001; Zhang et al. 2009; Lampayan et al. 2015). AWD is a water management technique in which, during the growing season, the field is cycled between being flooded and non-flooded where the water is left to drain away naturally (Zhang et al. 2009). Once the water level has decreased to 15–20 cm below the soil surface, the field is re-flooded and the next AWD cycle started (Lampayan et al. 2015). It has is reported that AWD can have varying impacts on yield, ranging from no impact on yield, a decrease in yield, or am increased yield compared to permanent flooding, with an increase in yield of 35% demonstrated in one study (Belder et al. 2004; Yang et al. 2007; Zhang et al. 2009; Carrijo et al. 2017). In addition to saving water, AWD has been reported to alter the accumulation of a range of elements in rice grains and shoots (Linquist et al. 2015; Norton et al. 2017a, b, 2019). For instance, it has been reported that AWD reduced grain Na^+^ compared to the plants grown under continuously flooded (CF) irrigation (Norton et al. 2017a, b).

Based on genetic variation, rice can be broadly classified into five subpopulations: *indica, aus, temperate japonica, tropical japonica* and *aromatic* (Garris et al. 2005; Zhao et al. 2010; Kim et al. 2016). The *aus* subpopulation of rice is considered to have rich genetic diversity with wide variation in abiotic stress resistance and is grown under a range of conditions from fully irrigated to upland (Glaszmann 1987; Ali et al. 2013). Several studies have been carried out to identify Na^+^ and K^+^ accumulation in rice, but most used either populations of *indica* or *japonica* rice (Kumar et al. 2015; Batayeva et al. 2018; Frouin et al. 2018; An et al. 2020). Additionally, a number of the screens were done using hydroponic systems where transpiration is low and is not representative of field conditions, and the imposed stress was either not gradual enough or too severe. Little attention has been devoted to investigating Na^+^ and K^+^ accumulation in *aus* rice shoots and grains under real field conditions. This study utilizes a population of rice derived from the *aus* subpopulation of rice that has been genotyped and can be used for GWA mapping. This population has previously been tested for seedling-stage salt tolerance using both a hydroponic and a soil-based system (Chen et al. 2020). In the current study, the population was grown under field conditions in Bangladesh in two consecutive dry seasons under both CF and AWD conditions. The aim of this study was to understand the impact that AWD has on Na^+^ and K^+^ accumulation in a wide collection of *aus* rice, to compare Na^+^ and K^+^ concentrations of field-grown plants to those screened for seedling-stage salt tolerance in hydroponic and soil systems, and to identify QTLs for Na^+^ and K^+^ accumulation and Na^+^/K^+^ ratio under field conditions. The longer-term goal is to identify stable QTLs that can be utilized to reduce Na^+^ concentration in rice, and therefore potentially breed rice that can be grown in more adverse environments.

## Materials and method

### Materials

The Bengal and Assam Aus Panel (BAAP) used in this study consists of 266 *aus* rice accessions and a small number of *indica* and *japonica* rice accessions (Table S1). The 266 *aus* rice accessions have been genotyped and a 2 million SNP database constructed (Norton et al. 2018).

### Field screening and Na and K concentration analysis

The BAAP population was grown in the field in Mymensingh, Bangladesh, in the Boro (dry season) of 2013 and 2014 under two irrigation methods; continuously flooded irrigation (CF) and the water saving technique alternate wetting and drying (AWD) as described in Norton et al. (2017a, 2018). Briefly, the rice seeds were initially sown in a nursery bed and then transplanted to the experimental field after 44 days in 2013 and 51 days in 2014. The seedlings were transplanted into eight experiment plots (four plots for AWD treatment and four plots for CF treatment); the experiment was set up as a random completed block design with four replicates. Plants were planted as two plants per hill with a distance of 20 cm between each hill in a row; there was a 20 cm gap between each row of 4 m length. The eight plots were flooded after transplanting. For CF treatment plots, the surface water was kept at a depth of between 2 and 5 cm above the soil surface during the vegetative and reproductive stage. For AWD treatment plots, plastic perforated tubes (pani pipe) were placed across the plots to monitor the water depth. This was to allow water to drain/percolate naturally from the AWD plots until the average depth of the water was 15 cm below the soil surface. At this point the plots were irrigated to bring the water depth to between 2 and 5 cm above the soil surface. AWD treatment was conducted from 14 days after transplanting until flowering. There were four AWD cycles, and once the fourth cycle had finished, the AWD and CF plots were maintained under flooded conditions during the flowering stage. The grains and shoots from every cultivar were harvested once the grains matured.

The electricity conductivity of the soil was measured using the soil water 1:5 extraction method (Visconti et al. 2010) and the exchangeable Na^+^ and K^+^ in the soil was measured using the ammonium acetate method (Allen et al. 1974). The particle size composition of the soil was 60.4% (± 2.2 (SD)) clay, 29.2% (± 2.1) silt, and 10.4% (± 1.5) sand. The pH of the soil was 6.6 (Norton et al. 2017a). The electrical conductivity (ECe) and Na^+^ concentration of the soil was 0.270 dS m^−1^ (± 0.04) and 0.108 mg g^−1^ (± 0.029), respectively, which indicated the soil was not under salt stress conditions. The soil K^+^ concentration was 0.068 mg g^−1^ (± 0.008). The day before transplanting of seedlings into the experimental plots, the plots were fertilized with 40 kg ha^−1^ nitrogen, 15 kg ha^−1^ phosphorus, 50 kg ha^−1^ potassium, 15 kg ha^−1^ sulfur and 3 kg ha^−1^ zinc. Further, 40 kg ha^−1^ nitrogen was supplied during the tiller stage and another 40 kg ha^−1^ nitrogen at the flowering stage (Norton et al. 2017a).

The Na^+^ and K^+^ concentrations in the shoots and grains of the rice were analyzed as described in Norton et al. (2017a). Briefly, rice grains were de-husked and oven dried (80 °C). A total of 0.2 g of dehusked grains were accurately weighed out and digested with nitric acid and hydrogen peroxide (Norton et al. 2012). Shoot samples were oven dried, powdered, accurately weighed (0.01 g) and digested using nitric acid and hydrogen peroxide on a block digester. Analysis of Na^+^ and K^+^ were performed by inductively coupled plasma—mass spectroscopy (ICP—MS). Trace element grade reagents were used for all digests. For quality control, replicates of certified reference material (Oriental basma tobacco leaves [INCT- OBTL- 5] and rice flour [NIST 1568b]) were used; blanks were also included. All samples and standards contained 10 µg L^−1^ indium as the internal standard.

### Genome-wide association mapping

GWA mapping was performed on all the *aus* cultivars from the BAAP using the PIQUE pipeline that first pre-processes genotype and phenotype and then conducts EMMAX analyses on each phenotype in parallel (Norton et al. 2018). SNPs with minor allele frequency (MAF) < 0.05 were filtered out and maximum per-SNP missing percentage was set at 5% for GWA. GWA mapping was conducted with traits and 2 million SNPs using an efficient mixed model (EMMA) controlling population structure and kinship, and a significance threshold of *p* < 0.0001 was used to determine significant SNPs, as described in Norton et al. (2018). The false discovery rate (FDR) of detected associations was estimated to calculate Benjamini–Hochberg adjusted probabilities (Benjamini and Hochberg 1995). A significance threshold of 10% FDR was used to identify putative SNP associations (McCouch et al. 2016) and highlighted in red in Manhattan plots. The FDR was subsequently used to identify QTLs.

### QTLs

After GWA, clump analysis was conducted to identify multiple significant SNPs (*p* < 0.0001) that represent a single QTL using PLINK (Purcell et al. 2007) command “–clump-*p*1 0.0001 –clump-*p*2 0.0001 –clump-*r*2 0.3 –clump-kb 243” as described in Norton et al. (2019). Singleton significant SNPs (*p* < 0.0001) were discarded if no other SNP were within the linkage disequilibrium (LD) decay window which had a *p* < 0.0001. Clump analysis was done for each trait separately. After clump analysis, the identified “clump” of all traits were put together to compare their positions; the clumps were merged into one if they were detected in different traits but in the same positions or in a 243 kb window. Note, 243 Kb is the genome-wide rate of linkage disequilibrium (LD) decay in the BAAP (Norton et al. 2018). After that, the FDR information of the significant SNPs in all QTLs was checked. Only QTLs which contained at least one significant (*p* < 0.0001) SNP that passed a 10% FDR were reported.

### Identification of candidate genes

For each QTL, a genomic distance of 243 kb around the peak SNP of each QTL was defined and the annotation of all genes in the regions were obtained from Rice Genome Annotation Project (RGAP) (http://rice.plantbiology.msu.edu/), release 7. For subsequent analysis, those genes annotated as “(retro) transposon,” “hypothetical,” or “unknown” were excluded from the identification of candidate gene analysis. To identify which of these genes were good candidates, gene information such as gene ontology classification and gene function in RGAP were examined, and then the positions of these genes (QTLs) were compared with genes previously reported to be involved in salt stress and/or sodium accumulation in rice from the literature.

### Haplotypes analysis

Four notable QTLs which were associated with multiple traits were further investigated. LD heatmaps surrounding the peaks of these QTLs were constructed using the R package “LDheatmap” using squared Pearson's correlation coefficient (*r*^2^). The gene expression profiles of all genes within the Local LD region were obtained from different studies (Walia et al. 2007; Shankar et al. 2016; Buti et al. 2019; Campbell et al. 2020) to confirm the validity of candidate genes. The SNPs which were significantly (*p* < 0.01) associated with the Na^+^ concentration and/or Na^+^/K^+^ ratio were extracted using PLINK. Non-synonymous and synonymous SNPs within the genes were identified based on the gene models of the annotated Nipponbare reference genome from RGAP using ANNOVAR software (Wang et al. 2010). The haplotypes based on visualization of the SNPs in the exon of the candidate genes in these four QTLs were determined and phenotypic response for the cultivars with each haplotype were observed.

### Statistical analysis

The cultivars which had phenotypic data in both AWD and CF conditions were used to do the two-way ANOVA using the software Minitab v.19 (State College, PA, United States). The boxplot, interplot, and Pearson correlation analysis were performed using the package “ggplot2” in R version 4.0. Principal component analysis for traits was performed using the package “factoextra” in R version 4.0. The plot of QTLs and candidate genes in different chromosomes were performed using the packages “plotrix” and “shape” in R version 4.0.

## Results

### Na^+^, K^+^ concentration and Na^+^/K^+^ ratio under AWD and CF

**Fig. 1:**
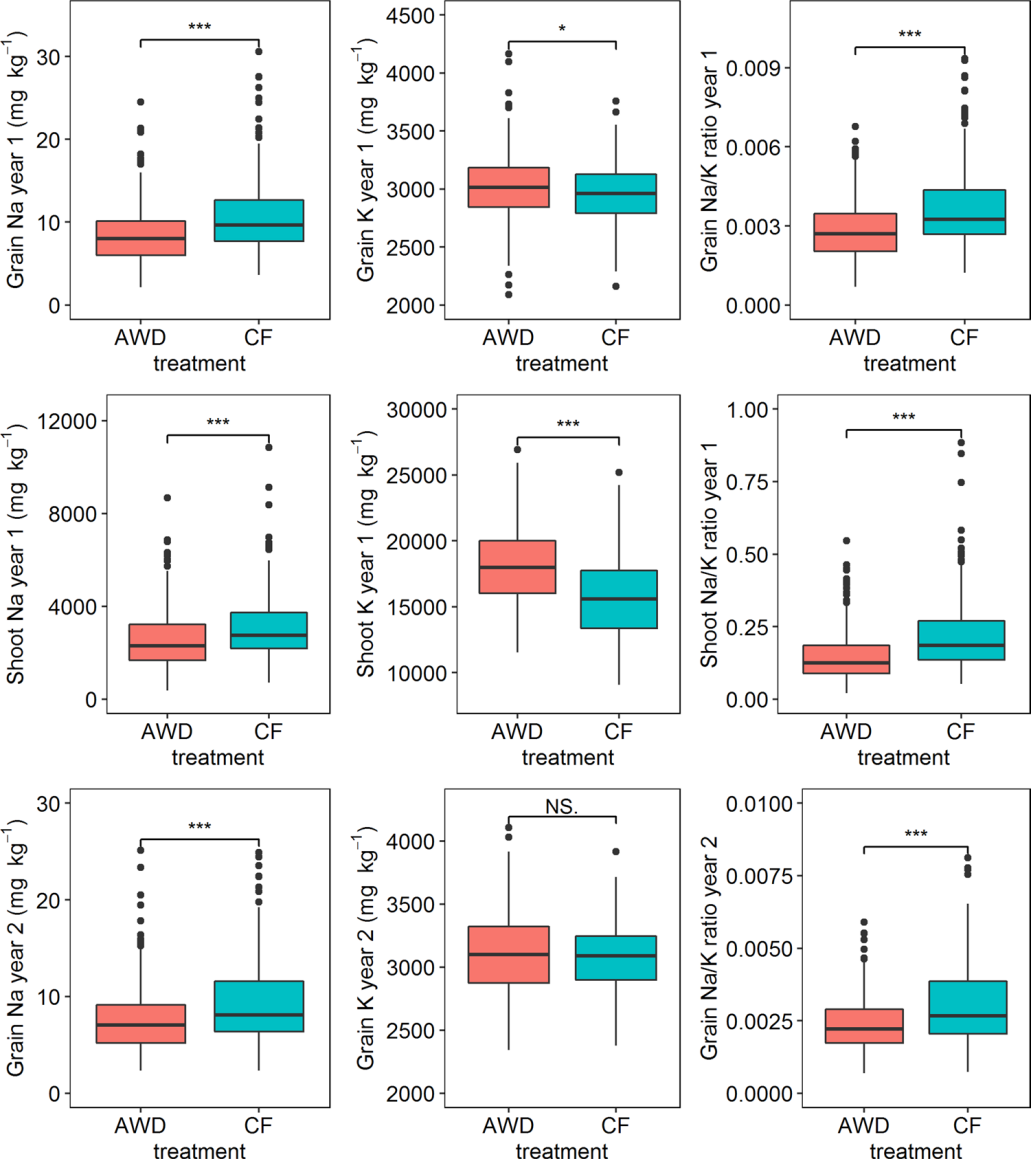
Na^+^, K^+^ concentration and Na^+^/K^+^ ratio in grains and shoots under AWD and CF, *p < 0.05, **p < 0.01, ***p < 0.001. Year 1 is year 2013, while year 2 is year 2014

There were significant differences between all measured traits, except grain K^+^ concentration in year 2, between the plants grown in AWD and CF. In both years grain and shoot Na^+^ concentration was lower in the plants grown under AWD (Fig. 1). In year 1, the average Na^+^ concentration of grains and shoots across the population in AWD treatment (8.6 mg kg^−1^ and 2556 mg kg^−1^, respectively) were significantly lower than that in CF treatment (10.9 mg kg^−1^ and 3120 mg kg^−1^, respectively). The average Na^+^/K^+^ ratio of grains and shoots in plants grown under AWD were 0.28% and 14.9%, respectively, which were significantly lower than plants grown under CF conditions (0.37% and 21.8%, respectively). In year 2, the average Na^+^ concentration and Na^+^/K^+^ ratio in the grains of plants grown under AWD (7.7 mg kg^−1^ and 0.24%, respectively) were significantly lower than the plants grown under CF conditions (9.5 mg kg^−1^ and 0.31%) (Table S2). There were significant genotype and treatment effects in all measured traits in both years while only the Na^+^ concentration and Na^+^/K^+^ ratio in grains in year 2 showed significant genotype × treatment interactions (Table 1). There were large variations between cultivars for Na^+^ concentration, K^+^ concentration and Na^+^/K^+^ ratio with the proportion of variation explained by genotype being between 27.8 and 41.7% (Table 1).

**Table 1:** The percentage contribution of genotypes, treatments (AWD and CF) and genotype × treatment interactions to the observed variation

Traits	Treatments	Genotypes	Genotypes × treatments
Grain Na^+^ year 1	4.4%***	40.7%***	NS
Grain K^+^ year 1	0.24%*	27.8%***	NS
Grain Na^+^/K^+^ year 1	5.2%***	38.6%***	NS
Shoot Na^+^ year 1	1.7%***	30.9%***	NS
Shoot K^+^ year 1	6.9%***	30.1%***	NS
Shoot Na^+^/K^+^ year 1	4.6%***	37.9%***	NS
Grain Na^+^ year 2	3.7%***	41.7%***	9.6%*
Grain K^+^ year 2	NS	27.5%***	NS
Grain Na^+^/K^+^ year 2	4.2%***	42.6%***	9.4%*

*p < 0.05, **p < 0.01, ***p < 0.001, NS, nonsignificant. Year 1 is year 2013, while year 2 is year 2014

Correlations between the corresponding traits under AWD and CF conditions were significant (*p* < 1 × 10^–8^) (Fig. S1), with the correlation coefficient (*r*) of 0.46 and 0.65 for Na^+^ concentration in shoots and grains, respectively, 0.48 and 0.44 for K^+^ concentration in shoots and grains, respectively, 0.63 and 0.65 for Na^+^/K^+^ ratio in shoots and grains, respectively, in year 1, and 0.68 for grain Na^+^, 0.4 for grain K^+^ and 0.7 for grain Na^+^/K^+^ ratio in year 2.

### PCA analysis

**Fig. 2:**
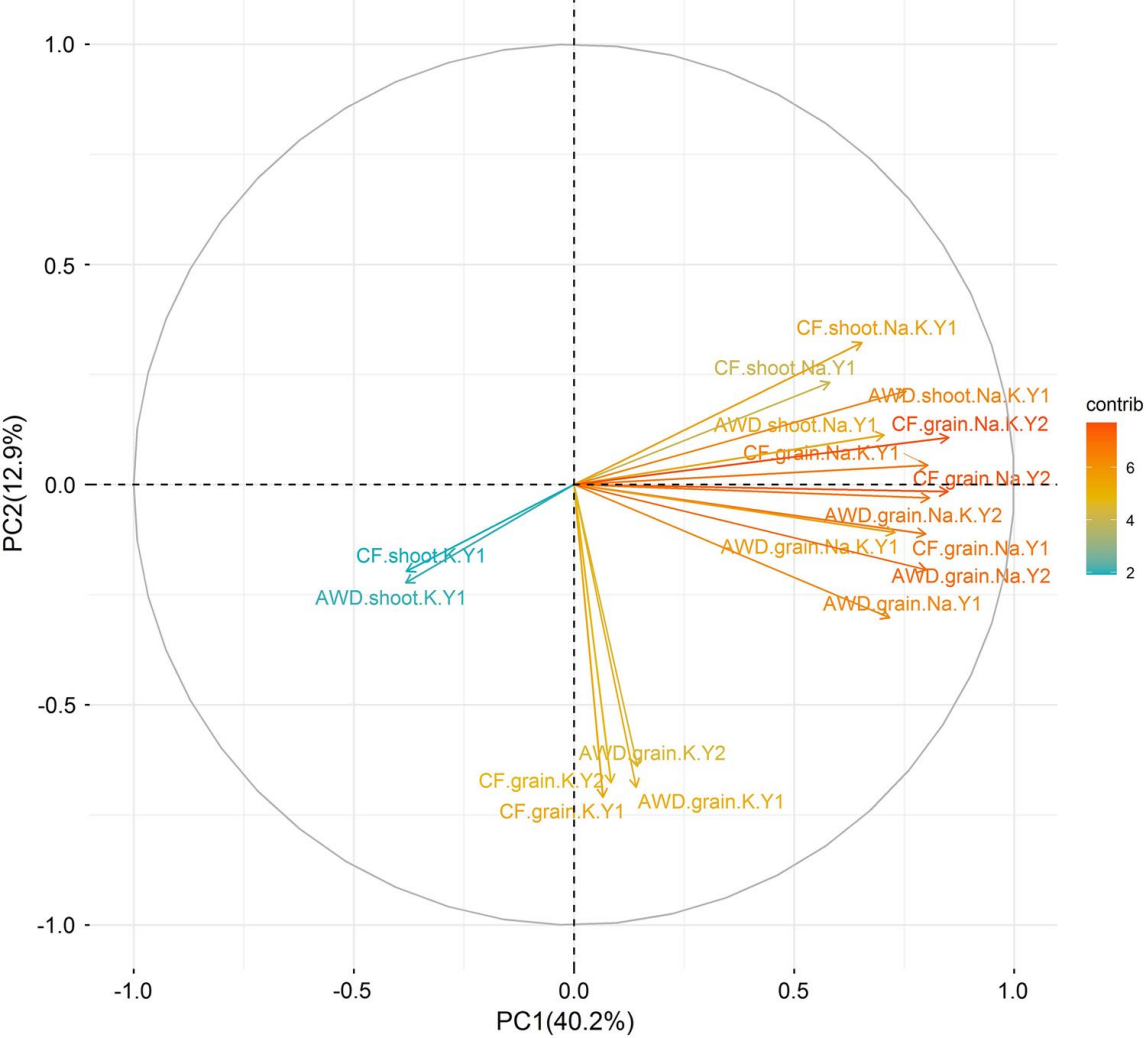
PCA for Na^+^, K^+^ concentration and Na^+^/K^+^ ratio under AWD and CF conditions. Y1: year 2013; Y2: year 2014. Contri means the contribution to the PCs

To investigate the relationship among trait variables and the factors underlying trait variation, principal component analysis (PCA) was performed for all 18 traits (Fig. 2; Fig. S2 and Table S3). PC1, PC2 and PC3 explained 40.2%, 12.9% and 10.3% of trait variance, respectively. Traits clearly occupied different spaces in the biplot with shoot K^+^ having negative values for both PC axis, grain K^+^ traits being slightly positive for PC1 and highly negative for PC2, while all Na^+^ traits including Na^+^/K^+^ ratio had very large positive values for PC1 and near zero for PC2. Among the Na^+^ traits, shoot traits (including Na^+^/K^+^ ratio) were the most positive for PC2.

### Aus subpopulation analysis

**Fig. 3:**
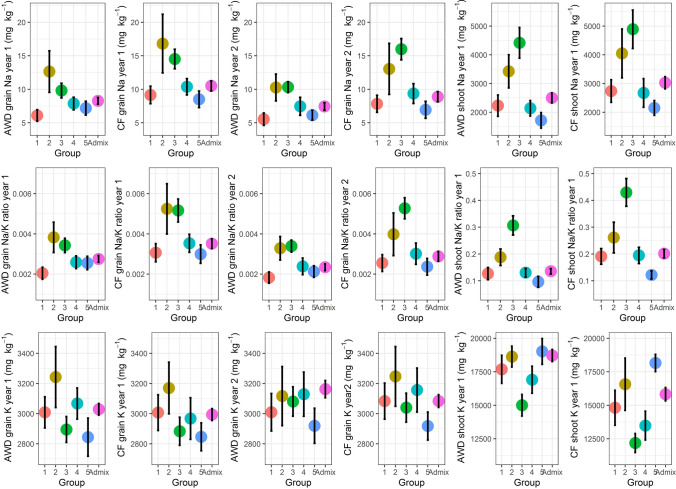
Range of Na^+^, K^+^ concentration and Na^+^/K^+^ ratio in grains and shoots variations under different BAAP groups. Bar is 95% confidence interval. BAAP groups as identified by Norton et al. (2018)

The BAAP population can be divided into five distinct groups by analysis of the population structure (Norton et al. 2018) (Table S1). One-way ANOVA showed that there were significant (*p* < 0.01) variations among these five groups in Na^+^, K^+^ concentration and Na^+^/K^+^ ratio (Fig. 3; Table S4). The proportion of variation explained by structure groups were between 6.9 and 47.3%, with the Na^+^/K^+^ ratio in shoots in year 1 having the largest values (AWD: 47.0%; CF: 47.3%), and the K^+^ concentration in grains in year 2 having the smallest values (AWD: 6.9%; CF: 7.3%) (Table S4). Comparison of the Na^+^, K^+^ concentration and Na^+^/K^+^ ratio in different structure groups showed that the cultivars in groups 2 and 3 were high for grain Na^+^ concentration and grain Na^+^/K^+^ ratio while groups 1, 4 and 5 were low. The cultivars in group 2 had high value for grain K^+^ concentration and group 5 had the lowest grain K^+^ concentration. For shoots, the cultivars in group 3 had the highest values in Na^+^ concentration and Na^+^/K^+^ ratio and the lowest values in K^+^ concentration, while group 5 had the lowest values in Na^+^ concentration and Na^+^/K^+^ ratio but the highest values in K^+^ concentration (Fig. 3).

### Comparison of Na^+^ and K^+^ concentration in the BAAP under different screening conditions

The BAAP has been screened for salt tolerance at the seedling stage under both a hydroponic (EC: 7  dS m^−1^) and a soil (EC: gradually increased from 2 to 9 dS m^−1^) system, and Na^+^ and K^+^ concentrations in shoots were measured (Chen et al. 2020). In this study, the Na^+^ and K^+^ concentrations in shoots and grains were measured in field conditions. Correlations between the Na^+^ and K^+^ concentration in different experiments (field, soil and hydroponic) were conducted (Table S5). The Na^+^ concentration in shoots and grains under AWD and CF conditions in field in both years positively correlated (*p* < 0.05) with Na^+^ concentration in shoots in hydroponics (*r* ranged from 0.18 to 0.32) and Na^+^ concentration in shoots in soil controlled environment experiment (*r* ranged from 0.18 to 0.32). There were no significant correlations for K^+^ concentration in plants grown in the field and in hydroponic environments, while the K^+^ concentration in shoots of plants grown in field under both AWD and CF conditions were significantly (*p* < 0.05) positively correlated with K^+^ concentration in shoots of plants grown in soil environment, with the correlation coefficient 0.20 and 0.18, respectively. For Na^+^/K^+^ ratio, except for the CF shoot Na^+^/K^+^ ratio in year 1, the Na^+^/K^+^ ratio in shoots and roots in field conditions positively correlated (*p* < 0.05) with Na^+^/K^+^ in shoots in hydroponics (r ranged from 0.15 to 0.31). The Na^+^/K^+^ ratio in shoots of plants grown under both CF and AWD in field were positively significantly (*p* < 0.01) correlated with shoots Na^+^/K^+^ ratio in plants grown in the soil-controlled environment experiment (*r* ranged from 0.27 to 0.30).

### GWA mapping for Na^+^ concentration and Na^+^ ratio

**Fig. 4:**
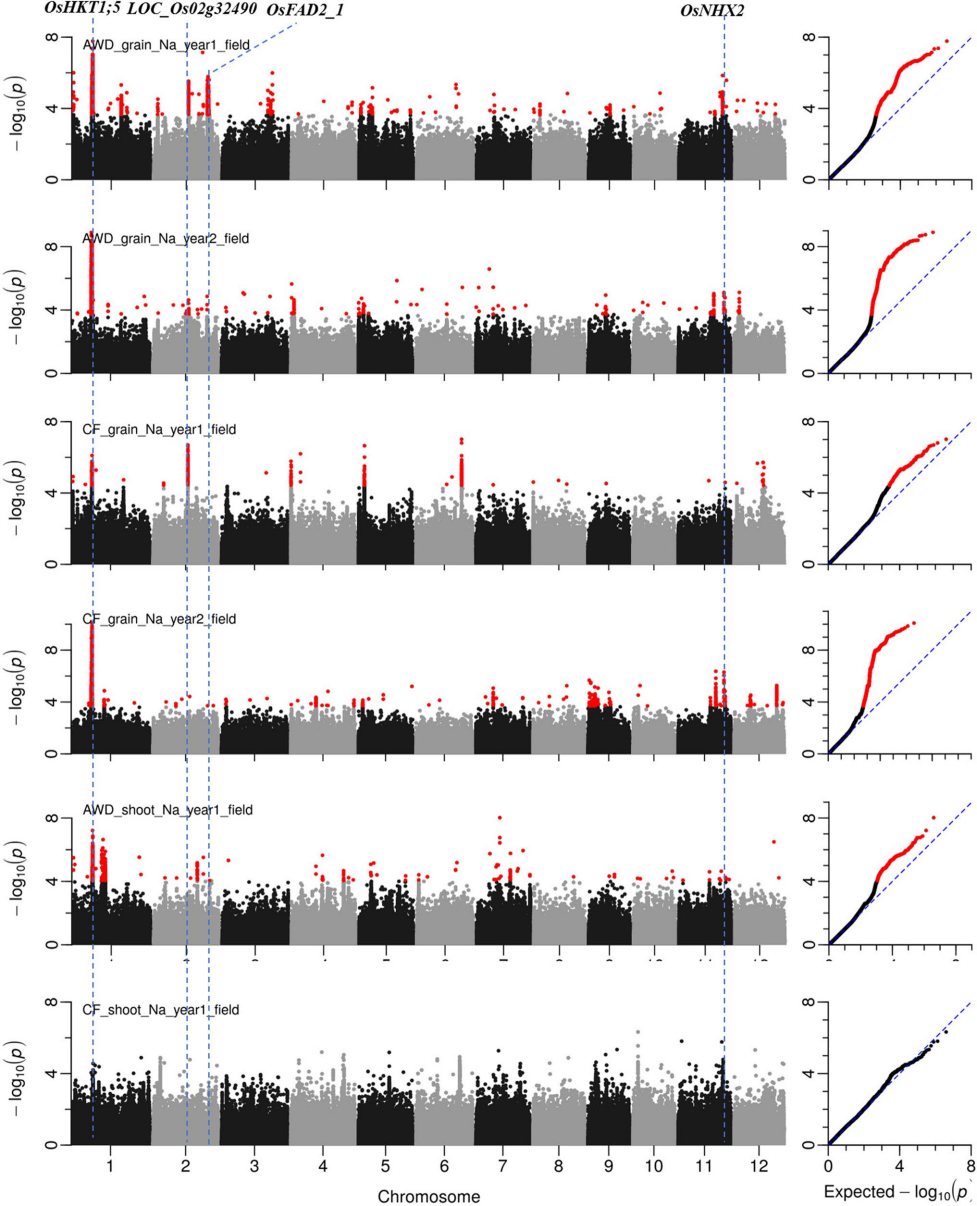
Manhattan plots from GWA mapping of Na^+^ concentration in grains and shoots under CF and AWD. Benjamini–Hochberg adjusted probabilities > 0.1 are highlighted in red dot. The diagonal blue line shown on QQ Plots represents 1:1 agreement between expected probability. Candidate genes for four notable QTLs are shown along on the top

GWA mapping was conducted on the BAAP to identify genomic loci controlling Na^+^ and K^+^ concentration and Na^+^/K^+^ ratio in grains and shoots in rice plants (Fig. 4, Fig. S3 and Fig. S4).

In total, 106 QTLs were associated with at least one of the 12 investigated traits (Table S6); 48 of the QTLs were associated with Na^+^ and/or Na^+^/K^+^ ratio in multiple years and/or multiple conditions (Table 2). Several QTLs were notable in terms of containing a large number of significant SNPs and with multiple traits associated with the QTL. The notable QTLs were on chromosome 1 around 11.45 Mb, which was detected in ten traits; on chromosome 11 between 25.63 and 26.06 Mb, which was detected in seven traits; on chromosome 2 between 19.20 and 19.25 Mb, which was detected in four traits; and on chromosome 2 between 29.66 and 30.14 Mb, which was detected in three traits. In addition, the QTLs on chromosome 5 (~ 3.34 Mb), chromosome 6 (~ 25.10 Mb) and the QTL on chromosome 11 (~ 20.75 Mb) were all associated with two traits.

**Table 2:** QTLs detected in multiple traits. The range of the QTL is presented as the QTL interval, and the position of the most statistically significant SNP for the trait association is given

Chr	QTL region	Grain Na^+^ year 1 AWD	Grain Na^+^ year 2 AWD	Grain Na^+^ year 1 CF	Grain Na^+^ year 2 CF	Shoot Na^+^ year 1 AWD	Shoot Na^+^ year 1 CF	Grain Na^+^/K^+^ ratio year 1 AWD	Grain Na^+^/K^+^ ratio year 2 AWD	Grain Na^+^/K^+^ ratio year 1 CF		Grain Na^+^/K^+^ ratio year 2 CF	Shoot Na^+^/K^+^ ratio year 1 AWD	Shoot Na^+^/K^+^ ratio year 1 CF
1	0.73–0.88	0.732				*0.875*								
1	0.86–1.06	*1.038*		1.002				1.038						
1	10.95–11.4	*11.057*	**11.154**		**11.158**			*11.206*	**11.154**		*11.205*	**11.158**	*11.259*	
1	11.17–11.59	**11.509**	**11.412**	***11.491***	**11.479**	**11.387**		**11.509**	**11.412**		*11.509*	**11.479**	**11.507**	
1	11.52–11.62								***11.581***			***11.522***		
1	13.17–13.17					13.166	13.166							
1	16.29–16.69					*16.508*							16.556	
1	17.01–17.36					***17.18***							17.18	
1	18.33–18.48					*18.407*							*18.404*	
1	20.3–20.3		20.299						20.298					
1	27.08–27.32	*27.154*						*27.3*						
1	30.16–30.33	30.234						***30.234***						
2	19.2–19.25	*19.221*		***19.209***				19.221			*19.209*			
2	23.99–24.12					*24.018*							**24.017**	
2	27.36–27.4	27.36				*27.387*								
2	29.59–29.64	*29.63*						*29.635*						
2	29.66–30.14	*29.898*	29.82					*29.888*						
2	29.95–30.35	*30.196*						*30.196*						
3	25.21–25.42	*25.205*						25.205						
4	0.07–0.28			*0.136*							***0.145***			
4	0.51–0.51		*0.508*						***0.508***					
4	5.36–5.36			***5.362***							***5.362***			
4	13.87–13.9				13.902							13.87		
4	28.7–28.82					28.712	28.817						*28.712*	
4	31.16–31.16	31.165						31.165						
5	3.15–3.38			***3.341***							***3.341***			
5	7.34–7.36	*7.356*						7.362						
5	16.96–17						*16.963*						*16.963*	*16.983*
6	21.44–21.63	*21.629*				21.443		21.629					21.443	
6	25.08–25.16			***25.104***							***25.106***			
7	9.54–9.54		*9.536*		*9.537*				***9.536***			*9.537*		
7	10.74–10.85					10.749								10.743
7	11.9–11.9					*11.899*							11.904	
7	12.67–12.84					**12.777**	12.838						***12.777***	12.838
7	18.52–18.64					*18.643*							*18.643*	
7	25.74–25.74				25.74							25.74		
8	0.12–0.20			0.182							0.182			
9	0.85–1.00				0.849							0.854		
9	1.56–1.57				*1.568*							1.568		
9	3.56–4.00				*3.784*							3.784		
9	14.24–14.42					14.236							*14.419*	
11	17.61–17.85				17.7							*17.7*	17.618	
11	19.64–19.67		*19.644*						19.643					
11	20.71–20.77				***20.753***							**20.753**		
11	21.04–21.08						21.081					21.038		
11	25.24–25.26		*25.243*		***25.243***							25.243		
11	25.63–26.06		25.809	25.873	25.83	25.632			25.833			25.877	25.632	
12	23.49–23.53				*23.498*							***23.498***		

Fonts indicate significance. Normal: 1e −5 <  = *p* value < 1e −4; Italic: 1e −6 <  = *p *value < 1e −5; Bolditalics: 1e −7 <  = *p* value < 1e −6; bold: *p* value < 1e −7

### Haplotypes analysis

**Fig. 5:**
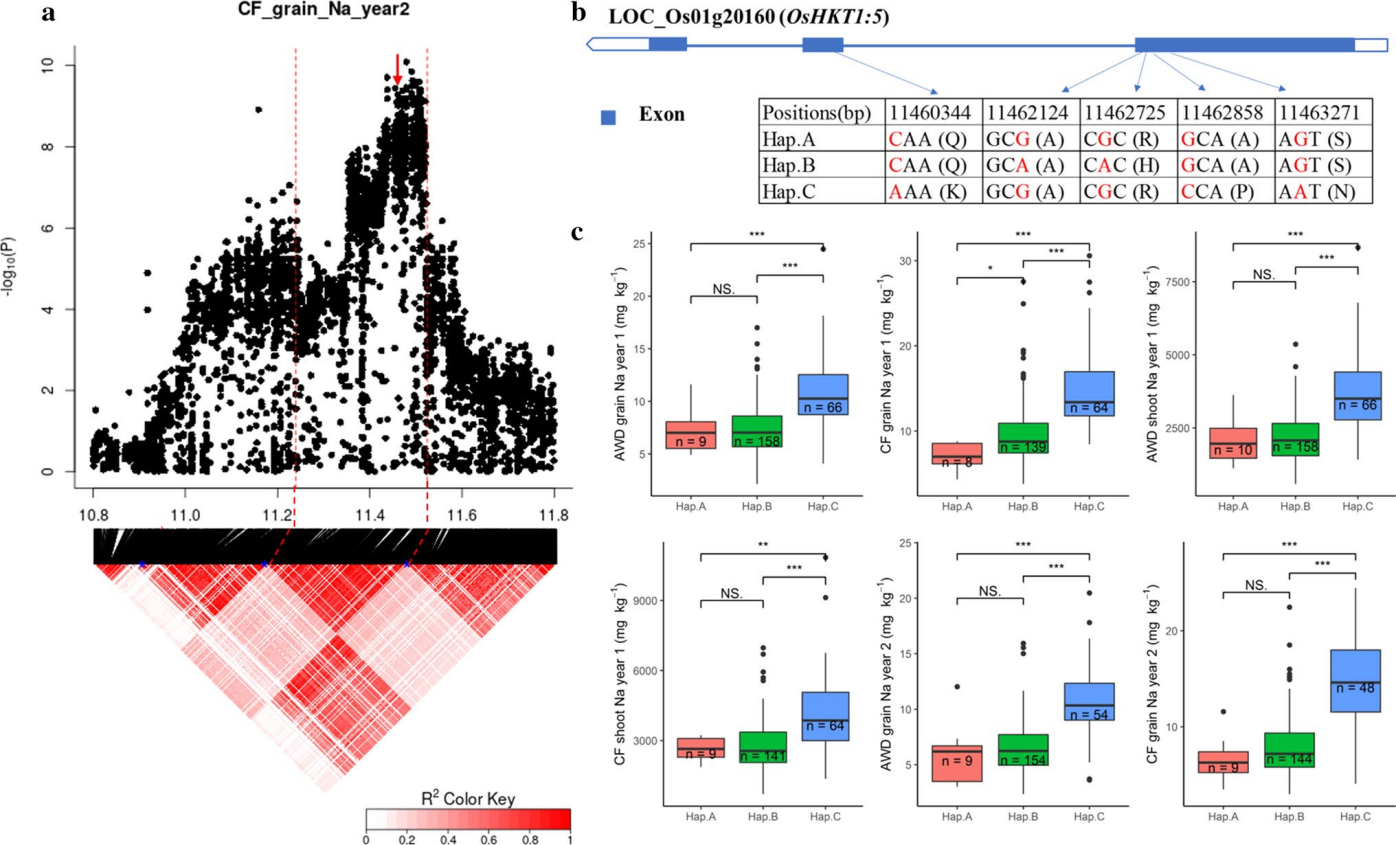
Significant association for Na^+^ concentration and Na^+^/K^+^ ratio in the grains and shoots on chromosome 1–11.45 Mb. **a** Local Manhattan plot (top) and LD heat map (bottom) of QTL on Chromosome 1, dash line represents the candidate regions between 11.25 and 11.52 Mb. Arrow indicates the position of candidate gene *LOC_Os01g20160* (*OsHKT1;5*); **b** the synonymous and nonsynonymous SNPs in the candidate gene *OsHKT1;5* significantly associated with Na^+^, and amino acid variations; **c** Na^+^ concentration in grains and shoots for indicated haplotypes of *OsHKT1;5*

The notable QTL on chromosome 1–11.45 Mb was associated with Na^+^ concentration and Na^+^/K^+^ ratio in shoot and grains in both years and both water treatments (Fig. 4 and Fig. S4). The QTL region was estimated to be from 11.24 to 11.52 Mb by pairwise LD correlations (Fig. 5a). There are 25 genes in this QTL region based on RGAP (Table S7). Among them, 15 genes have previously been shown to be either significantly differentially expressed due to salt treatment or to have different expression between cultivars (Walia et al. 2007; Shankar et al. 2016; Buti et al. 2019; Campbell et al. 2020) (Table S7). *LOC_Os01g20160* (*OsHKT1; 5*) was selected as the candidate gene as the transcript levels of this gene in 91 rice accessions are highly divergent, and RNA expression of M103 (salt sensitive cultivar) and Agami (salt tolerance cultivar) were significantly (*p* < 0.001) different under both control and salt stress conditions during the panicle initiation stage (Table S7). A total of 34 SNPs in this candidate gene are listed in the BAAP 2 million SNP database, and five of them are in the exons of this gene, four of which were nonsynonymous SNPs. The physical positions (based from the start of the chromosome) of these four nonsynonymous SNPs were at 11,460,344 bp (C/A polymorphism), 11,462,725 bp (G/A polymorphism), 11,462,858 bp (G/C polymorphism) and 11,463,271 bp (G/A polymorphism), which resulted in the amino acid substitutions from Gln (Q) to Lys (K), Arg (R) to His (H), Ala (A) to Pro (P) and Ser (S) to Asn (N), respectively. In addition, a synonymous polymorphism was at 11,462,124 bp (G/A) (Fig. 5b). The cultivars in this study carried three haplotypes for these five SNPs, A (*n* = 10), B (*n* = 158) and C (*n* = 66) (Fig. 5b). The cultivars carrying A and B haplotypes showed significantly lower grain and shoot Na^+^ concentration and Na^+^/K^+^ ratio (Fig. 5c and Fig. S5) while K^+^ concentrations were not significantly different (data not presented).

**Fig. 6:**
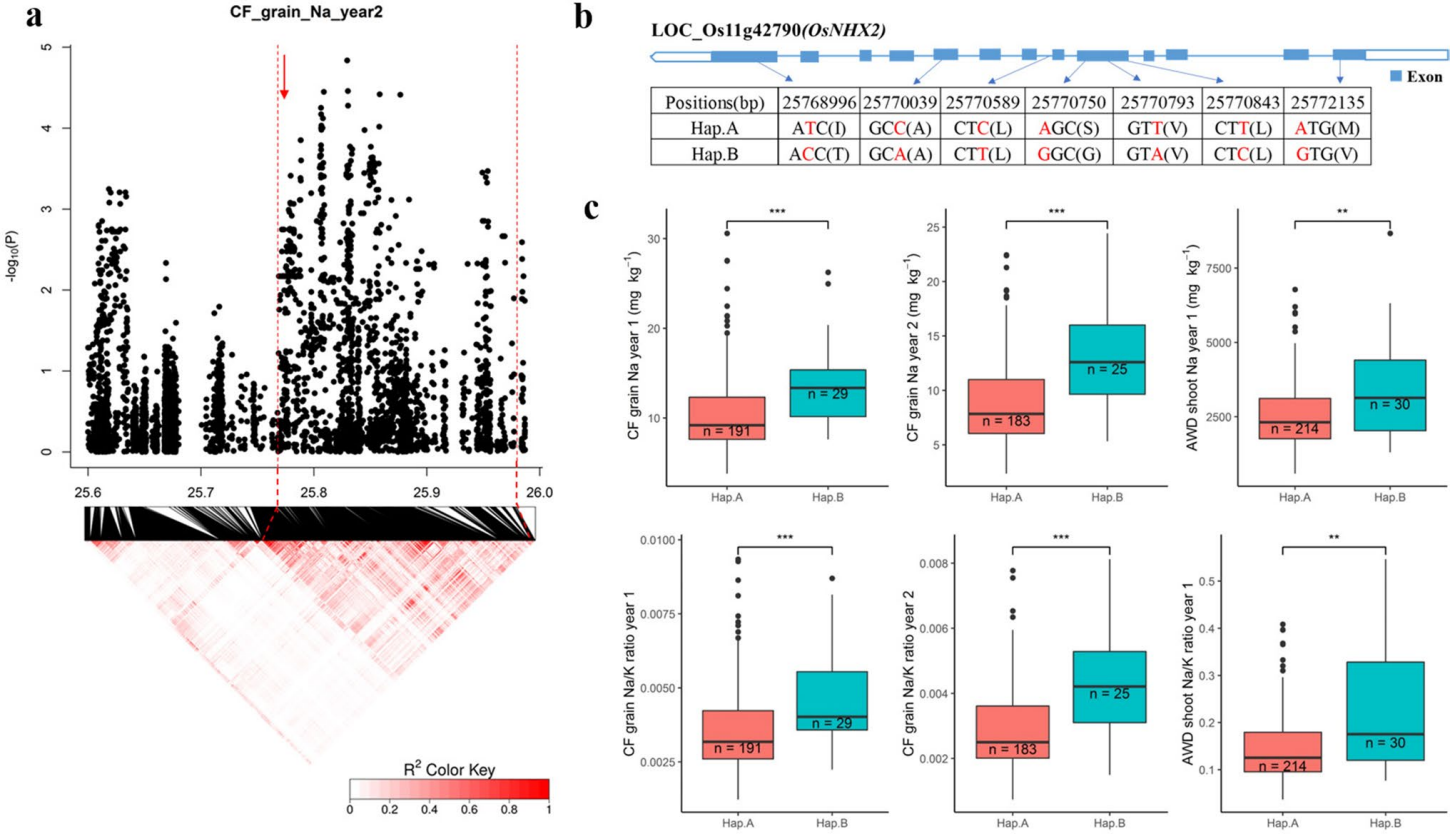
Significant association for Na^+^ concentration and Na^+^/K^+^ ratio in the grains and shoots on chromosome 11–25.80 Mb. **a** Local Manhattan plot (top) and LD heat map (bottom) of QTL on Chromosome 11, dash line represents the candidate regions between 25.75 and 25.98 Mb. Arrow indicates the position of candidate gene *LOC_Os11g42790* (*OsNHX2*) (Positioned at 25.77 Mb*)*; **b** the synonymous and nonsynonymous SNPs in the candidate gene *OsNHX2* significantly associated with the Na^+^, and amino acid variations; **c** Na^+^ concentration and Na^+^/K^+^ ratio in grains for indicated haplotypes of *OsNHX2*

A strong QTL on chromosome 11–25.80 Mb was associated with Na^+^ concentration and Na^+^/K^+^ ratio in grains and shoots (Fig. 4 and Fig. S4). The QTL region was estimated to be from 25.76 to 25.98 Mb (Fig. 6). There were 21 genes in this QTL region. Among them, 12 genes have previously been shown to be either significantly differentially expressed due to salt treatment or to have different expression between cultivars (Walia et al. 2007; Shankar et al. 2016; Buti et al. 2019; Campbell et al. 2020) (Table S8). *LOC_Os11g42790* (*OsNHX2*) which is annotated as a monovalent cation: proton antiporter was selected as the candidate gene in this region (Fig. 6a). The expression of this gene in cultivars N22 and Vialone Nano in salt stress was significantly (*p* < 0.001) upregulated, and the RNA expression was significantly (*p* < 0.001) different between M103 and Agami under control condition during the panicle initiation stage (Walia et al. 2007; Shankar et al. 2016; Buti et al. 2019) (Table S8). Seven SNPs in the exons of the gene were significantly associated with traits, three of which were nonsynonymous SNPs. These three nonsynonymous SNPs were at the positions 25,768,996 bp (T/C polymorphism), 25,770,750 bp (A/G polymorphism), and 25,772,135 bp (A/G polymorphism), which resulted in a change at amino acid from Ile (I) to Thr (T), Ser (S) to Gly (G) and Met (M) to Val (V), respectively. Four synonymous polymorphisms were at 25,770,039 bp (C/A), 25,770,589 bp (C/T), 25,770,793 bp (T/A) and 25,770,843 bp (T/C) (Fig. 6b). The cultivars carried two haplotypes for these seven SNPs, the common (*n* = 191) A haplotype and rarer (*n* = 29) B haplotype (Fig. 6b, c). The Na^+^ concentration in grains in these two haplotypes were significantly different. The cultivars carrying the A haplotypes had lower grain Na^+^ content than cultivars carrying the B haplotype (Fig. 6c and Fig. S6).

**Fig. 7:**
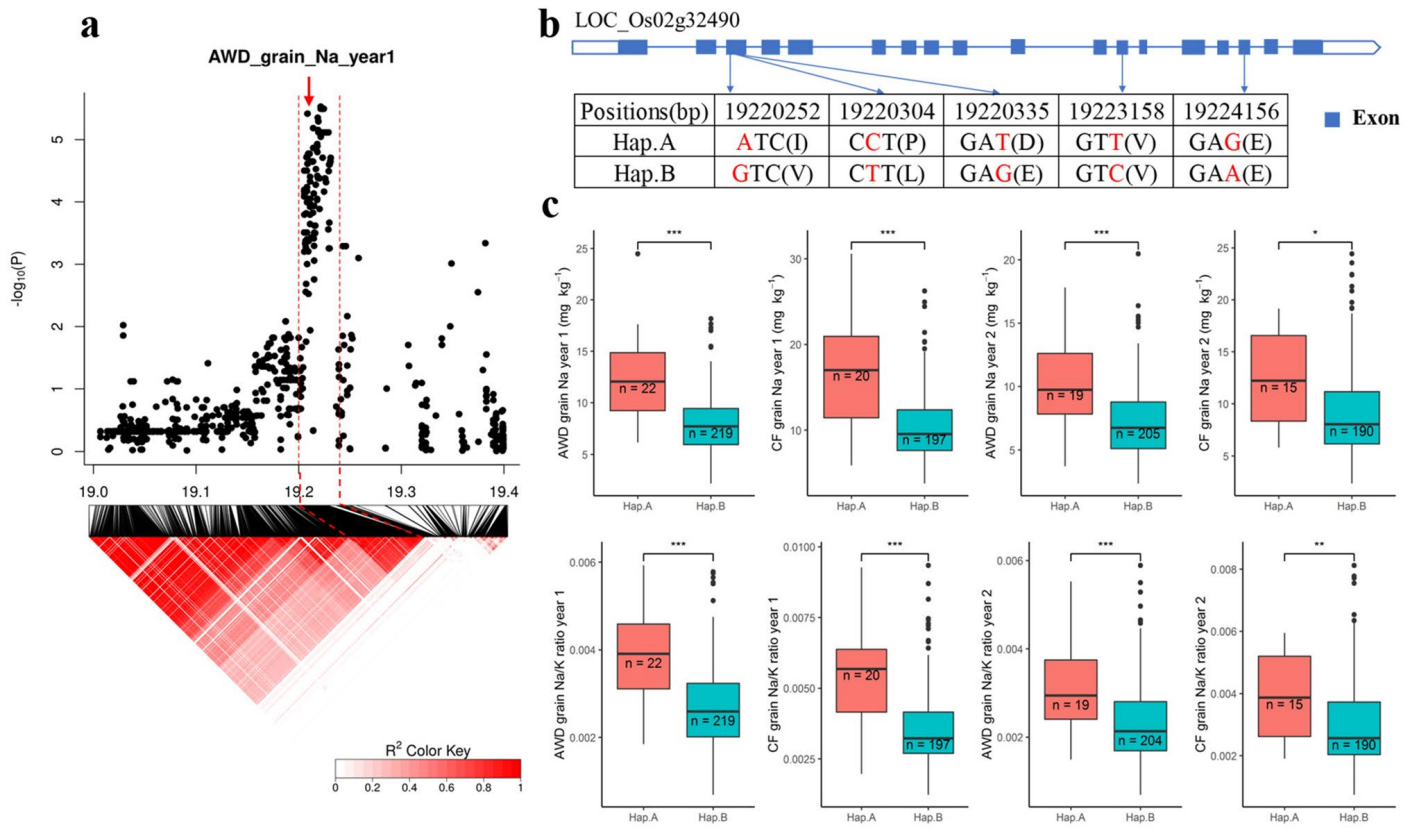
Significant association for Na^+^ concentration and Na^+^/K^+^ ratio in the grains on chromosome 2–19.20 Mb. **a** Local Manhattan plot (top) and LD heat map (bottom) of QTL on Chromosome 2, dash line represents the candidate regions between 19.20 and 19.25 Mb, Arrow indicates the position of candidate gene *LOC_Os02g32490*; **b** the synonymous and nonsynonymous SNPs in the candidate gene *LOC_Os02g32490* significantly associated with the Na^+^, and amino acid variations; **c** Na^+^ concentration in grains for indicated haplotypes of *LOC_Os02g32490*

Another notable QTL is on chromosome 2–19.20 Mb which was associated with Na^+^ concentration and Na^+^/K^+^ ratio in grains in both AWD and CF conditions (Fig. 4 and Fig. S4). The QTL region was estimated to be from 19.20 to 19.24 Mb by using pairwise LD correlations (Fig. 7a). There are five genes in this local LD region. Among them, four genes have previously been shown to be either significantly differentially expressed due to salt treatment or to have different expression between cultivars (Walia et al. 2007; Shankar et al. 2016; Buti et al. 2019; Campbell et al. 2020) (Table S9). The gene *LOC_Os02g32490* which is annotated as AMP-binding enzyme was proposed as the candidate gene based on the RNA expression. The RNA levels in a salt sensitive cultivar (Vialone Nano) were significantly (*p* < 0.001) upregulated under salt stress. Additionally the transcript levels of *LOC_Os02g32490* in 91 rice accessions was highly divergent under control conditions, and the RNA expression of M103 and Agami were significantly (*p* < 0.001) different under control conditions during the panicle initiation stage (Walia et al. 2007; Buti et al. 2019; Campbell et al. 2020) (Table S9). There were 22 SNPs in *LOC_Os02g32490* in the BAAP; five of them were SNPs in the exons which are significantly associated with Na^+^, while three of them were nonsynonymous SNPs. These three nonsynonymous SNPs were at the positions 19,220,252 bp (A /G polymorphism), 19,220,304 bp (C/T polymorphism), and 19,220,335 bp (T/G polymorphism) which resulted in a change at amino acid from Ile (I) to Val (V), Pro (P) to Leu (L), and Asp (D) to Glu (E). Two synonymous polymorphisms were 19,223,158 bp (T/C) and 19,224,156 bp (G/A) (Fig. 7b). The cultivars carried two haplotypes for these five SNPs, a rare (*n* = 22) A haplotype and a common (*n* = 197) B haplotype (Fig. 7b, c). The Na^+^ concentration in grains in these two haplotypes were significantly different. The cultivars carrying the A haplotype had higher Na^+^ concentration and Na^+^/K^+^ ratio in grains than cultivars carrying the B haplotype in both years and under both water treatments (Fig. 7c).

**Fig. 8:**
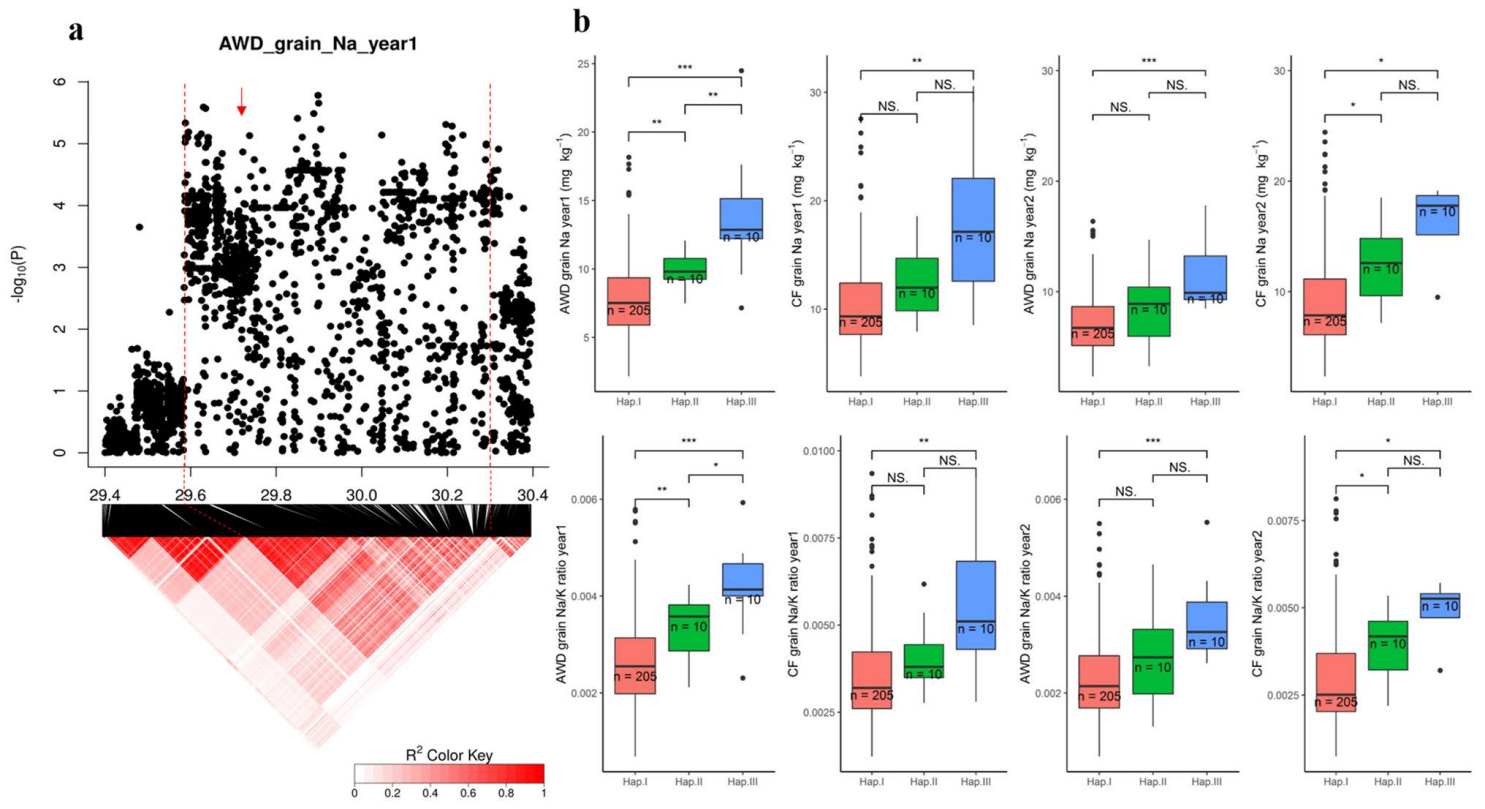
Significant association for Na^+^ concentration and Na^+^/K^+^ ratio in the grains on chromosome 2–30.0 Mb. **a** Local Manhattan plot (top) and LD heat map (bottom) of QTL on Chromosome 2, dash line represents the candidate region between 29.59 and 30.30 Mb, Arrow indicates the position of candidate gene *LOC_Os02g48560* (*OsFAD2_1*) (Positioned at 29.73 Mb); **b** Na^+^ concentration and Na^+^/K^+^ ratio in grains for indicated haplotypes of *LOC_Os02g48560*

Another notable QTL on Chromosome 2–29.9 Mb was significantly associated with grain Na^+^ concentration and Na^+^/K^+^ ratio in plants grown under AWD irrigation (Fig. 4; Fig. S4). The QTL region was estimated to be from 29.59 to 30.30 Mb by using pairwise LD correlations (Fig. 8a); this region contains 100 annotated genes. Among them, 85 genes have previously been shown to be either significantly differentially expressed due to salt treatment or to have different expression between cultivars (Walia et al. 2007; Shankar et al. 2016; Buti et al. 2019; Campbell et al. 2020) (Table S10). Based on genes’ function and their RNA expression (Table S8), gene *LOC_Os02g48560* (*OsFAD2_1*) annotated as fatty acid desaturase was proposed as the candidate gene in this locus. The RNA level in *LOC_Os02g48560* was significantly (*p* < 0.05) downregulated under salt stress in both sensitive and tolerant cultivars (Buti et al. 2019), and the RNA expression of this gene in M103 and Agami were significantly (*p* < 0.001) different under control conditions during the panicle initiation stage (Walia et al. 2007) (Table S10). There were 20 SNPs in the *LOC_Os02g48560* (*OsFAD2_1*) and no nonsynonymous SNPs were found. The cultivars in the BAAP carried three haplotypes among these 20 SNPs, I (*n* = 205), II (*n* = 10) and III (*n* = 10). The Na^+^ concentration and Na^+^/K^+^ ratio in grains in I haplotype were significantly lower than that in III haplotype (Fig. 8b).

## Discussion

### Comparison of Na^+^ and K^+^ concentration and Na^+^/K^+^ ratio in plants grown under AWD and CF conditions

Several studies have reported that the accumulation of a range of elements were different under AWD irrigation compared to CF (Chou et al. 2016; Norton et al. 2017b, 2019). Grain Na^+^ and K^+^ concentration both decreased under AWD irrigation in a panel of 22 cultivars, of which 19 are in the BAAP (Norton et al. 2017b). In this study, more BAAP cultivars (*n* = 284) were used to test the impact of AWD irrigation on Na^+^ and K^+^ concentration. The results also showed that the grain Na^+^ and shoot Na^+^ concentration across the population decreased in AWD, while the grain K^+^ and shoot K^+^ concentration across the population increased under AWD irrigation (except for the grain K^+^ in year 2) (Fig. 1). Under CF conditions there is more leaching of applied fertilizer (such as N, P, K), compared to AWD (Islam et al. 2016). This phenomenon of leaching in CF could be the reason why the plants uptake less K^+^ when grown under CF compared to AWD. The results of Na^+^ and K^+^ concentration in plants grown under AWD and CF may suggest that AWD could be used as a way of reducing Na^+^ accumulation (and increasing K^+^) in rice and therefore growing rice in mildly saline environments. Furthermore, there was high genotypic contribution (27.5–41.7%) for the observed variation for Na^+^ and K^+^ traits while there was a low treatment (water management) contribution (0.24–6.9%). A small number of genotype by treatment interactions were found and some QTLs were detected in specific water treatments. These results indicate that the mechanism of both Na^+^ and K^+^ transport for plants under AWD and CF may be different but there are likely to be common mechanisms as well. The PC biplots (Fig. 2) showed that the Na^+^ accumulation and Na^+^/K^+^ ratio in shoots and grains were assigned together but were different with K^+^ accumulation, and the K^+^ accumulation in shoots and grains were assigned into different directions. These results indicate that the Na^+^ accumulation and Na^+^/K^+^ ratio are different to K^+^ accumulation, and also that the K^+^ accumulation in shoots and in grains are regulated differently.

### Comparison of Na^+^ concentration and Na^+^/K^+^ ratio in different BAAP groups

The BAAP population can be divided into five distinct groups based on population structure (Norton et al. 2018). There were differences in the accumulation of Na^+^ and K^+^ for plants in these different groups (Fig. 3). Groups 1, 3 and 5 are predominantly from Bangladesh while groups 2 and 4 are predominantly from India (Norton et al. 2018). There were 28 cultivars in group 3, of which 20 cultivars have the term “boro” in their names and are all from Bangladesh. The term “boro” refers to a growing season in Bangladesh and the Assam region during December–May, and also to the rice cultivars grown during this season, which are referred to as winter rice (GRiSP 2013; Khush 1997). Group 3 in this study showed high Na^+^ concentration and Na^+^/K^+^ ratio in grains and shoots but low K^+^ concentration in grains and shoots (Fig. 3). A total of 18 out of 29 cultivars in group 5 have the term “aus” in their names. “Aus” refers to a growing season in Bangladesh and Assam during April–August, and also to the cultivars grown in this season (GRiSP 2013; Travis et al. 2015). These “aus” rice cultivars are broadcast, insensitive to photoperiod and are drought tolerant, and are referred to as summer rice (Khush 1997). Group 5 in this study showed low Na^+^ concentration and Na^+^/K^+^ ratio in grains and shoots while they were the highest in K^+^ in shoots. These results indicate that the rice cultivars used to growing in the summer season have lower Na^+^ accumulation and higher K^+^ accumulation than the cultivars growing in the winter season.

The salt tolerance of these five groups in hydroponics and soil (under controlled environmental conditions) has previously been estimated (Chen et al. 2020). Group 3 cultivars, which have high shoot and grain Na^+^ when grown in the field (Fig. 3), were previously identified as having high shoot Na^+^ concentration and high salt sensitivity in both soil and hydroponic salt tolerance screens of seedlings. Similarly, cultivars in groups 1, 4 and 5, which have low Na^+^ concentration and Na^+^/K^+^ ratio in this study, had low shoot Na^+^ and were tolerant to Na^+^ in the seedling screen (Chen et al. 2020). These results highlight the reliability of the tolerance tests and the value of measuring Na^+^ concentration in field grown plants in the absence of salt stress.

### QTLs

**Fig. 9:**
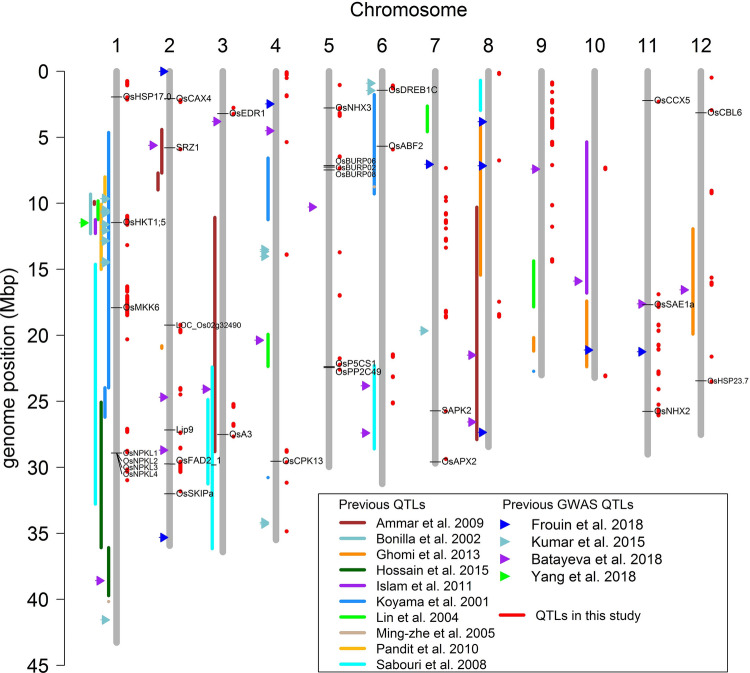
Locations of the QTLs for Na^+^, K^+^ concentration and Na^+^/K^+^ ratio detected in this study and in previously studies. Candidate genes identified in this study were indicated by horizontal lines

A total of 106 QTLs were associated with the 12 traits in this study, and 48 of them were detected in multiple years and conditions. A comparison of QTLs detected in this study with the previously published QTLs for Na^+^, K^+^ concentration and Na^+^/K^+^ ratio showed that 32 QTLs were co-located (Fig. 9). For example, the QTL on chromosome 1 between 11.169 and 11.59 Mb, which was associated with 10 traits in this study, co-located with the *Saltol,* the major QTL (~ 9.3–12.3 Mb) associated with Na^+^ concentration and Na^+^/K^+^ ratio (Koyama et al. 2001; Pandit et al. 2010; Islam et al. 2011; Kumar et al. 2015). This QTL has previously been identified as being associated with Na^+^ accumulation under normal field conditions (Yang et al. 2018). The QTL on chromosome 1 between 27.08 and 27.32 Mb was found to be associated with two traits here which were co-located with a previous QTL (Sabouri and Sabouri 2008; Hossain et al. 2015). The QTL on chromosome 11 between 17.61 and 17.85 Mb, which was associated with three traits in this study was co-located with a QTL in a previous GWA study for Na^+^/K^+^ ratio (Batayeva et al. 2018). These results suggest the QTLs identified in this study are real and stable. There are still a number of notable QTLs identified in this study which have not been reported previously and were identified as being associated with multiple traits/conditions. For instance, the QTL on chromosome 2–19.2 Mb was associated with four traits, the QTL on chromosome 2–29.9 Mb was associated with two traits and the QTL on chromosome 7–12.78 Mb was associated with four traits. As these QTLs were detected in multiple traits it suggests they are stable, and as they have not been previously detected it could suggest that they are specific for *aus* rice cultivars.

Some of the QTLs detected here appear to be tissue specific but some are not. There were several QTLs for Na^+^ concentration detected in both grains and shoots, while some QTLs were only detected in either grains or shoots (Table 2). The QTL on chromosome 1 between 11.24 and 11.59 Mb, the QTL on chromosome 4–27.36 Mb and QTL on chromosome 11 between 25.63 and 26.06 Mb were detected in both shoot Na^+^ and grain Na^+^. In contrast, the Na^+^ QTLs on chromosome 1 between 0.86 and 1.06 Mb, on chromosome 2–19.20 Mb and on chromosome 2–29.80 Mb were detected only in grains, and the Na^+^ QTLs on chromosome 1–13.17 Mb and on chromosome 4–28.2 Mb were only detected in shoots (Table 2).

A comparison of the106 QTLs detected here in field conditions with the QTLs for salt tolerance identified under salt stress in hydroponic and soil systems in the same BAAP population (Chen et al. 2020) showed 16 and 13 of the QTLs were co-located with the QTLs for salt tolerance detected in the hydroponic and soil system, respectively (Table S6). Four of these QTLs were found in all three experiments. The first is the QTL on chromosome 1 between 16.29 and 16.69 Mb which was detected to be associated with shoot Na^+^ concentration and Na^+^/K^+^ ratio in field conditions, with relative root length (root length in salinity stress *versus* root length in control conditions) in the hydroponic environment and with shoot Na^+^ concentration in the soil environment. The second QTL was on chromosome 4–29.61 Mb and was found to be associated with shoot Na^+^/K^+^ ratio in field conditions, with salt injury score in the hydroponic environment, and with relative shoot length in the soil environment. The third QTL was on chromosome 7–9.54 Mb and was found to be associated with grain Na^+^ concentration in field conditions, with shoot Na^+^ concentration in the hydroponic environment and with shoot Na^+^/K^+^ ratio in the soil environment. The fourth QTL was on chromosome 12 between 15.99 and 16.17 Mb and was identified as being associated with grain Na^+^ concentration in the field, with salt injury score in the hydroponic environment and with relative tiller number (tiller number in salinity stress *versus* tiller number in control condition) in the soil environment. The traits measured in the field were under normal conditions without salt stress imposed while the traits in hydroponic and soil system were in moderate salt stress conditions. The QTLs which were detected in salt stress and non-salt stress conditions may indicate that these QTLs regulate the Na^+^, K^+^ accumulation and not just expressed in salt stress.

### Candidate genes

A total of 31 candidate genes which have been previously reported to be involved in salt stress and/or sodium accumulation were found to be within ± 243 Kb of the peak SNP (the global LD decay of the BAAP population (Norton et al. 2018)) (Fig. 9 and Table S11). Several candidate genes are ion transporters. The most notable one, gene *OsHKT1;5*, a Na^+^ transporter, was found under the QTL on chromosome 1 between 11.24 and 11.59 Mb. *OsHKT1;5* is a Na^+^ selective transporter involved in Na^+^ and K^+^ homeostasis (Ren et al. 2005), and is also reported to be involved in Na^+^ accumulation in normal field condition (Yang et al. 2018). Here, three haplotypes were found in *aus* cultivars based on five SNPs in the exon significantly associated with Na^+^ concentration. The cultivars carrying A and B haplotypes had lower Na^+^ concentration in grains and shoots than the cultivars carrying the C haplotype. The haplotype A is the rarest haplotype in the BAAP and is only present in 10 of the cultivars. Four non-synonymous SNPs with the amino acid substitutions (S2N, P140A, H184R, Q429K) were presented. These four amino acid substitutions were reported in the study of Yang et al. (2018) from 529 rice cultivars, and they indicated the H184R is likely the key substitution that causes functional variation in *OsHKT1;5* (Yang et al. 2018). The P140A alteration was reported to increase the probability of *OsHKT1;5* phosphorylation (Negrão et al. 2013). It was reported that the haplotypes of *OsHKT1;5* in Goria (which was identified as a salt tolerant cultivar under salt stress in hydroponic and soil experiments) in the BAAP were identical to one accession called Nona Bokra (IRS_313-7736) (salt tolerance check), while different to Pokkali (IRIS_313-8244) (salt tolerance check) and another accession called Nona Bora (CX273) based on 92 SNPs (Chen et al. 2020).

*OsNHX3* and *OsNHX2*, the monovalent cation: proton antiporters, having a role of compartmentation of Na^+^ and K^+^ accumulated in the cytoplasm into vacuoles, were found in the QTL region located on chromosome 5–2.80 Mb, and in the QTL on chromosome 11–25.80 Mb, respectively. Expression analysis of the *OsNHX3* gene in rice indicates that it is highly expressed in the flag leaf and blades (Fukuda et al. 2011). Sodium chloride stress resulted in an increase in transcript abundance in the roots and shoots of *OsNHX3* (Fukuda et al. 2011). Expression analysis of *OsNHX2* in rice indicates that it is highly expressed in the flag leaf, sheaths, culms and panicles (Fukuda et al. 2011). Sodium chloride stress resulted in an increase in transcript abundance in the roots and shoots (Fukuda et al. 2011). *OsNHX2* can enhance salt tolerance in rice through more effective accumulation of toxic Na^+^ in leaf mesophyll and bundle sheath cells (Teng et al. 2017). In this study, seven SNPs in the exons of *OsNHX2* were found to be significantly associated with Na^+^ concentration, in which two were non-synonymous SNPs. Two haplotypes of these seven polymorphisms were found in the BAAP; the B haplotype significantly decreased the Na^+^ concentration in grains but studies of allelic variation on this gene have not been conducted.

For the QTL on chromosome 2–19.2 Mb, five genes were found in the QTL local LD region (19.20–19.24 Mb). To identify which of these genes are potential candidate genes, the expression profiles of these genes were explored. Previously it has been demonstrated that the RNA expression levels of *LOC_Os02g32490 (ACS1)* were significantly upregulated under salt and osmotic stress (Buti et al. 2019). The RNA expression between salt tolerance and salt sensitive cultivars were also significant under control conditions (Walia et al. 2007). Additionally, there was a significant difference in RNA expression for this gene between rice cultivars from the *indica* and *japonica* subpopulations (Campbell et al. 2020). *LOC_Os02g32490* is annotated as an AMP-binding enzyme from the RGAP and was reported as encoding acetyl-CoA synthetase1 (Kaya et al. 2017). Two haplotypes were found in five SNPs in the exon of this gene in the BAAP, and the B haplotype had low grain Na^+^ and Na^+^/K^+^ ratio (Fig. 7). The function between this gene and Na^+^ accumulation needs to be explored.

The QTL on chromosome 2–29.9 Mb detected in this study was also found to be associated with Na^+^ concentration and Na^+^/K^+^ ratio under salt stress in hydroponic experiments (Chen et al. 2020). *LOC_Os02g48560 *(*OsFAD2_1*), a fatty acid desaturase was proposed as the potential candidate gene for this QTL. Excess salt causes ion toxicity inside the cell and creates hyperosmotic stress in plants, thereby causing secondary stresses, such as oxidative stress resulting from the accumulation of reactive oxygen species (ROS) (Zhu 2001). Stress acclimating plants respond to abiotic and biotic stress by remodeling membrane fluidity and by releasing α-linolenic acid from membrane lipids. The modification of membrane fluidity is mediated by changes in unsaturated fatty acid levels, a function provided in part by the regulated activity of fatty acid desaturases (Zhu 2001, Zhiguo et al. 2019). Fatty acid desaturases play an important role in fatty acid metabolism and maintenance of the biological function of membranes in plant cells (Singh et al. 2002; Kaya et al. 2017; Sui et al. 2017). It has been suggested that omega-3 fatty acid desaturase overexpression can enhance the tolerance of early seedlings to salinity stress in tomato plants, by maintaining the integrity of the membrane system (Wang et al. 2014). Sui et al. (2018) reported salt stress markedly changed the activity of a fatty acid desaturase and fatty acid composition in peanut plants. RNA expression of *LOC_Os02g48560 *(*OsFAD2_1*) under salt and osmotic stress was significantly downregulated (Buti et al. 2019). These results indicate that fatty acid desaturase may also play an important role in rice in response to stress, but it must be noted that no nonsynonymous SNPs within this gene were detected in the BAAP.

### Combined low Na haplotypes

It is interesting to note that for all the QTLs discussed in all cases, the most common haplotypes are for low Na^+^ concentration. To identify if any accessions have multiple low haplotypes, the haplotype variations of four candidate genes in four notable QTLs were analyzed. Among 266 *aus* rice accessions in the BAAP, 130 of them carry low Na^+^ haplotypes across all four QTLs. The cultivars BOWALIA 2, T 65, T 1, and AUS 125, which were reported to be more salt tolerant than the check genotype (Pokkali) under salt stress in hydroponic and soil experiments (Chen et al. 2020) were all carrying the low Na^+^ haplotypes (Table S1). The results show that most of the accessions in the BAAP already have low Na^+^ haplotypes for breeding and these accessions can be used for confirming the presence of low Na^+^ rice breeding materials, and can also be used for breeding lower Na^+^ accumulation in rice.

To determine if these common alleles of the four candidate genes in the *aus* rice are also common in the *indica* and *japonica* rice*,* the haplotypes analysis tool RiveVarMap (Zhao et al. 2015) which included the cultivars from the Rice Diversity Panel were employed (Table S12). The common alleles of *OsHKT1;5* (Hap. B) and *OsFAD2_1* (Hap. I) in the *aus* rice are the rare alleles in both *indica* and *japonica* rice. The common allele of *OsNHX2* (Hap. A) in the *aus* rice is the common allele in the *indica* and *japonica* rice. The common allele (Hap. B) of *LOC_Os02g32490* in the *aus* rice is the common allele of *japonica* rice while it is the rare allele of *indica* rice.

### Na^+^ concentration under salt stress and non-salt stress

The Na^+^ concentration of the BAAP was previously evaluated under salt stress in hydroponic and soil-based environments (Chen et al. 2020). In this study, the Na^+^ concentration of the BAAP was measured in a non-salt stress field environment. There were significant correlations of Na^+^ concentration under salt stress and non-salt stress conditions (Table S5). The salt tolerant cultivars (BOWALIA 2, T 65, T 1 and AUS 125), identified under salt stress in hydroponic and soil experiments (Chen et al. 2020), were carrying the low Na^+^ haplotypes reported in the current study. Additionally, several QTLs for Na^+^ concentration identified in non-salt stress conditions were co-located with the QTLs identified in salt stress conditions. For instance, the notable QTL on chromosome 1–11.45 Mb (same chromosome position as the well know QTL *Saltol)* which was found to be associated with Na^+^ concentration in this study (non-salt stress in field) was also identified as being associated with Na^+^ in the hydroponic experiment (salt stress) (Table S6). This QTL was also associated with Na^+^ accumulation in 529 rice cultivars in field experiments without salt stress imposed (Yang et al. 2018). The gene *OsHKT1;5*, regulating K^+^/Na^+^ homeostasis under salt stress (Ren et al. 2005), was also detected for Na^+^ accumulation in non-salt stress (Yang et al. 2018). These results indicate that the mechanisms and genes regulating Na^+^ concentration in the absence of salt stress are relevant for salt tolerance.

## Conclusion

In this study, we evaluated the Na, ^+^ K^+^ concentration and Na^+^/K^+^ ratio in grains and shoots of the BAAP population with ~ 300 cultivars under the AWD and CF water treatments over 2 years. The Na^+^ concentration and Na^+^/K^+^ ratio of plants grown under AWD conditions were significantly lower than plants grown under CF, while K^+^ concentration in plants grown under AWD were significantly higher than that in CF (except for the grain K^+^ in year 2). The Na^+^ concentration in shoots and grains in the field were positively significantly correlated with Na^+^ concentration in shoots under salt stress in both hydroponics and in soil experiments. A GWA study was conducted for these 18 traits and a total of 106 QTLs were identified, with 48 of them being found to be associated with multiple traits. The haplotypes analysis of four candidate genes in four notable QTLs was investigated. The low Na^+^ accumulation cultivars and QTLs for Na^+^ identified in this study could provide some useful information for future studies on breeding of low Na^+^ accumulation rice.

## Supplementary Information

Below is the link to the electronic supplementary material.

Supplementary file 1 (XLSX 185 kb)

Supplementary Fig. S1. The Pearson correlation of corresponding traits under AWD and CF in year 1 and year 2. The shaded area represents 95% confidence intervals. Year 1 is year 2013, while year 2 is year 2014. (JPG 823 kb)

Supplementary Fig. S2 PCA (PC1 and PC3) for Na+, K+ concentration and Na+/K+ ratio under AWD and CF conditions. Y1: year 2013; Y2: year 2014. Contri means the contribution to the PCs. (JPG 749 kb)

Supplementary Fig. S3. Genome-wide association for K+ concentration in grains and shoots under CF and AWD. Benjamini-Hochberg adjusted probabilities > 0.1 are highlighted in red dot. The diagonal blue line shown on QQ Plots represents 1:1 agreement between expected probability. (JPG 383 kb)

Supplementary Fig. S4 Genome-wide association for Na+/K+ ratio in grains and shoots under CF and AWD. Benjamini-Hochberg adjusted probabilities > 0.1 are highlighted in red dot. The diagonal blue line shown on QQ Plots represents 1:1 agreement between expected probability. (JPG 431 kb)

Supplementary Fig. S5. Na+/K+ ratio in grains and shoots for indicated haplotypes of OsHKT1; 5. (JPG 1039 kb)

Supplementary Fig. S7. Na+ concentration and Na+/K+ ratio in grains and shoots for indicated haplotypes of OsNHX2. (JPG 862 kb)

## Data Availability

The phenotype data used for the genome-wide association mapping is available in supplementary Table 1. The SNP dataset is available as a project called “BAAP” in the SNP-Seek database and on the Harvard DataVerse as a dataset called “Genome Wide Association mapping of grain and straw biomass traits in the rice Bengal and Assam Aus Panel (BAAP).”
