# Supplementary figures and images for: Genome-wide association mapping of sodium and potassium concentration in rice grains and shoots under alternate wetting and drying and continuously flooded irrigation
Source: Theor Appl Genet. 2021 May 4;134(7):2315–34. doi: 10.1007/s00122-021-03828-9 (PMC8263461; doi:10.1007/s00122-021-03828-9)

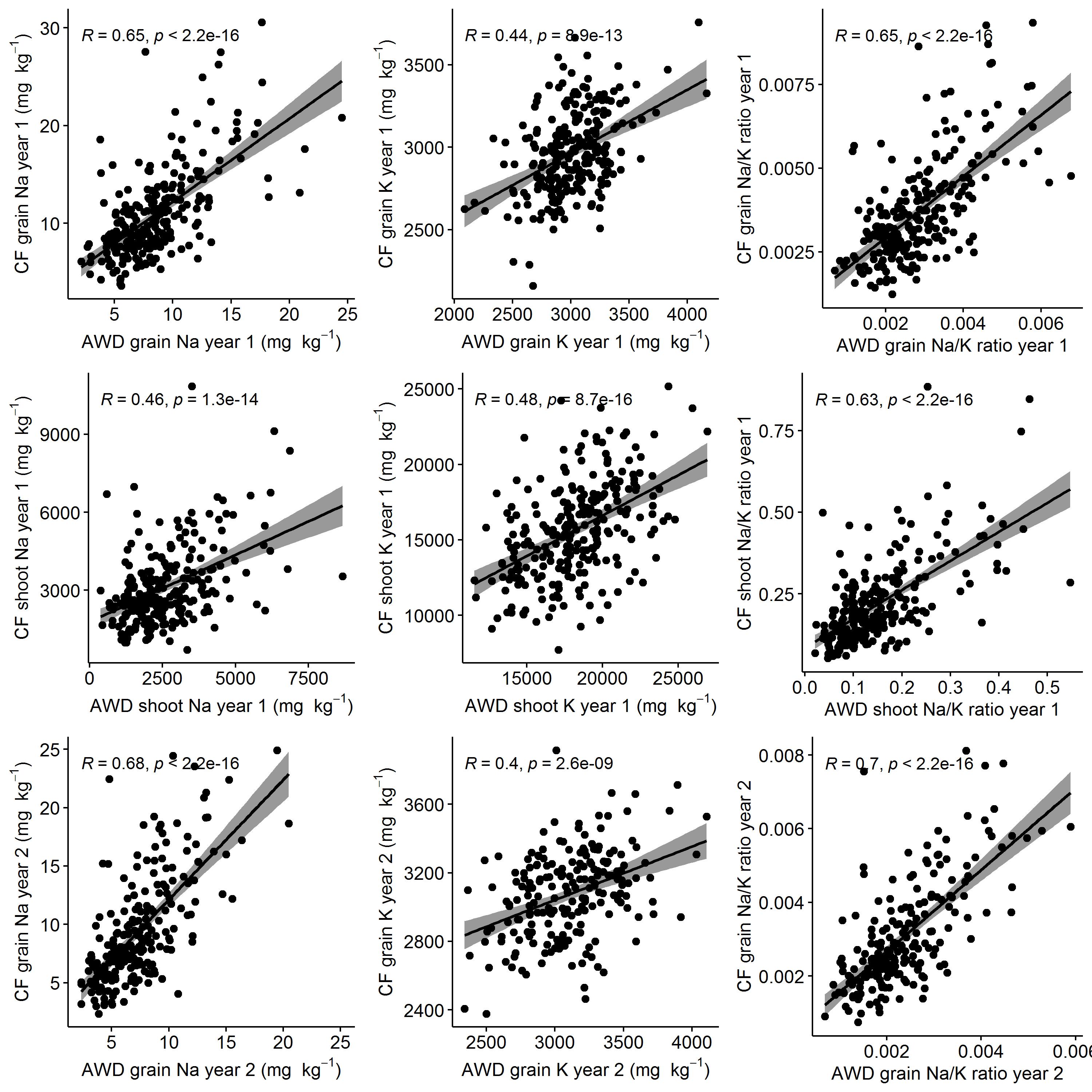

Supplement: Supplementary file 2 — Supplementary Fig. S1. The Pearson correlation of corresponding traits under AWD and CF in year 1 and year 2. The shaded area represents 95% confidence intervals. Year 1 is year 2013, while year 2 is year 2014. (JPG 823 kb) [file 122_2021_3828_MOESM2_ESM.jpg]

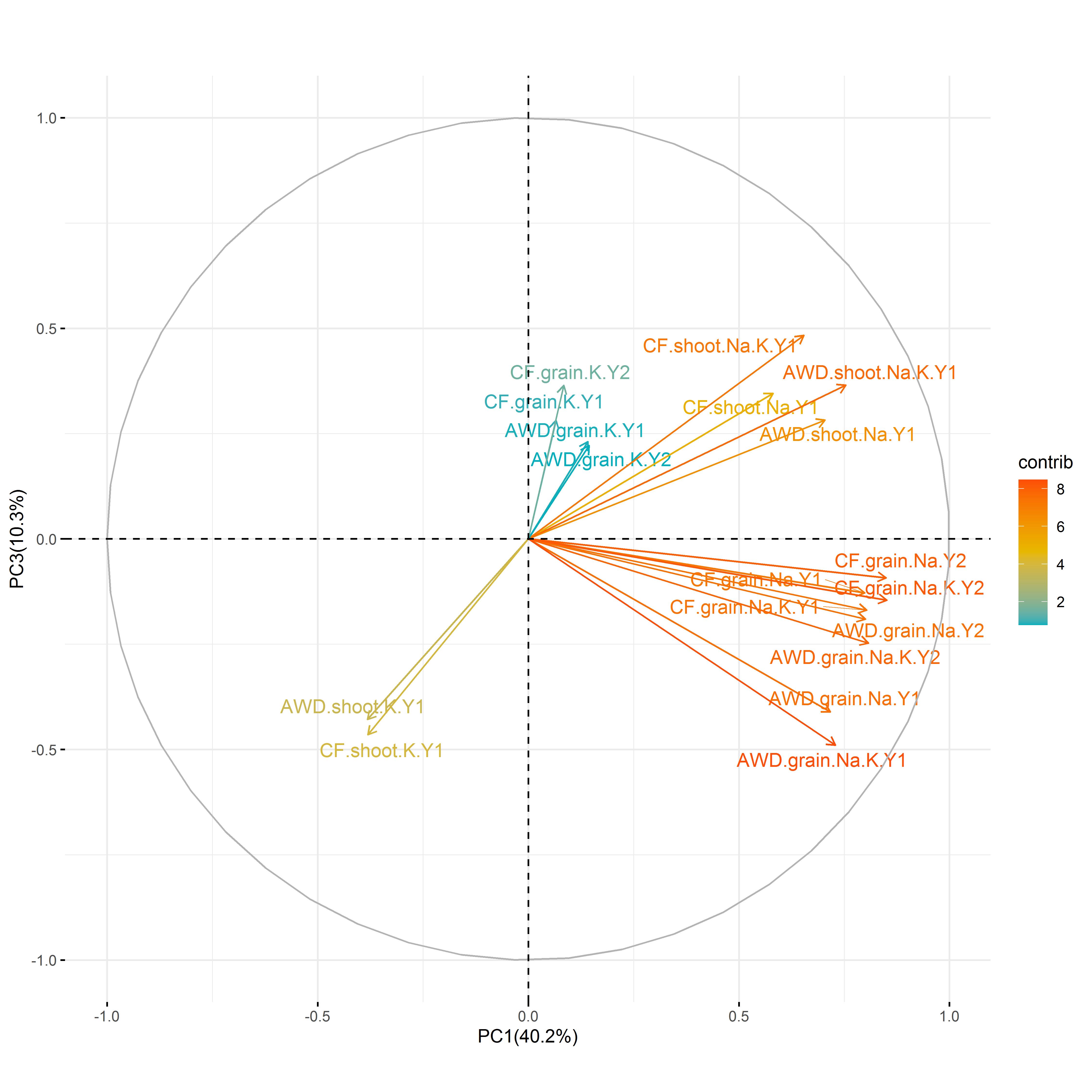

Supplement: Supplementary file 3 — Supplementary Fig. S2 PCA (PC1 and PC3) for Na+, K+ concentration and Na+/K+ ratio under AWD and CF conditions. Y1: year 2013; Y2: year 2014. Contri means the contribution to the PCs. (JPG 749 kb) [file 122_2021_3828_MOESM3_ESM.jpg]

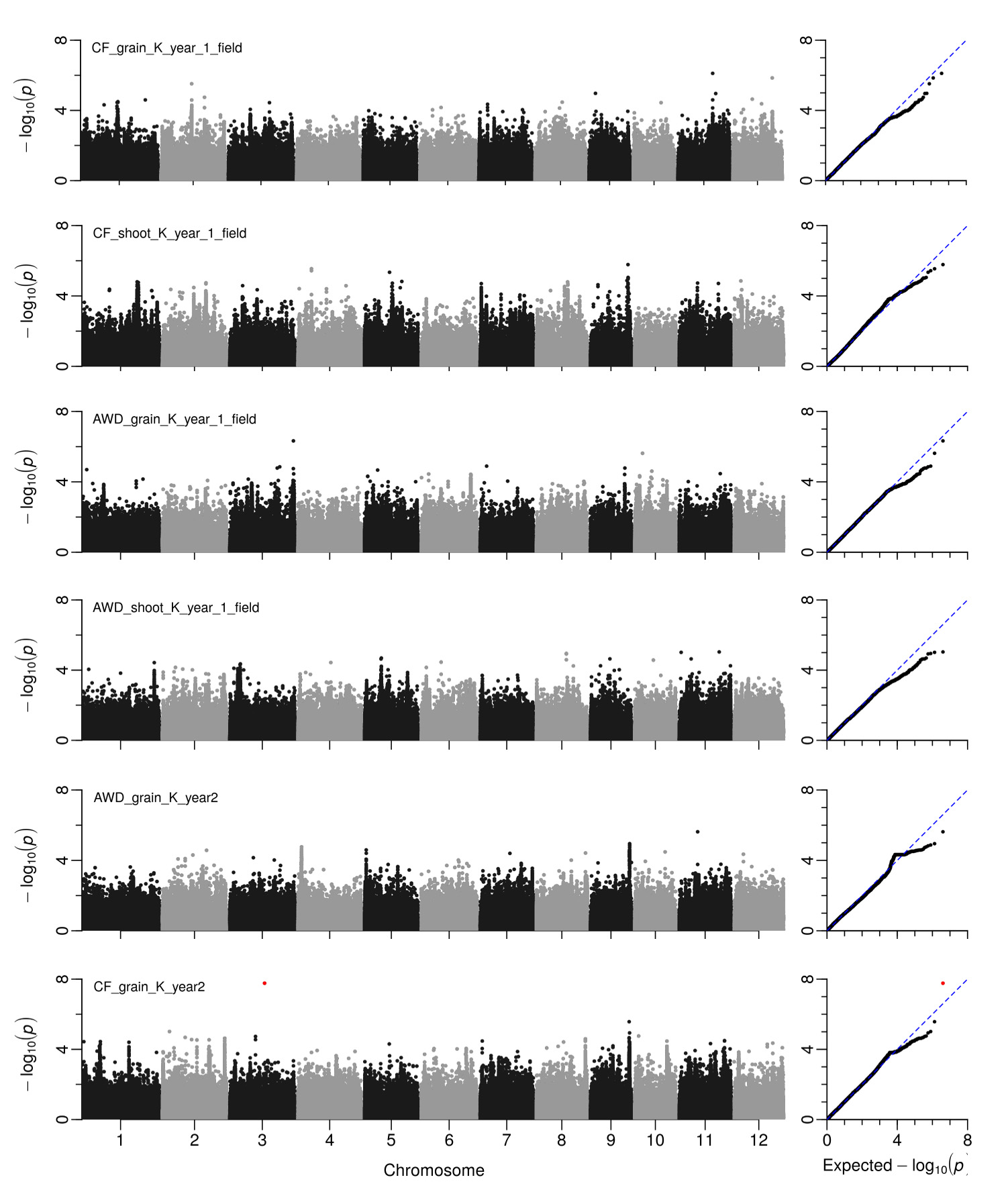

Supplement: Supplementary file 4 — Supplementary Fig. S3. Genome-wide association for K+ concentration in grains and shoots under CF and AWD. Benjamini-Hochberg adjusted probabilities > 0.1 are highlighted in red dot. The diagonal blue line shown on QQ Plots represents 1:1 agreement between expected probability. (JPG 383 kb) [file 122_2021_3828_MOESM4_ESM.jpg]

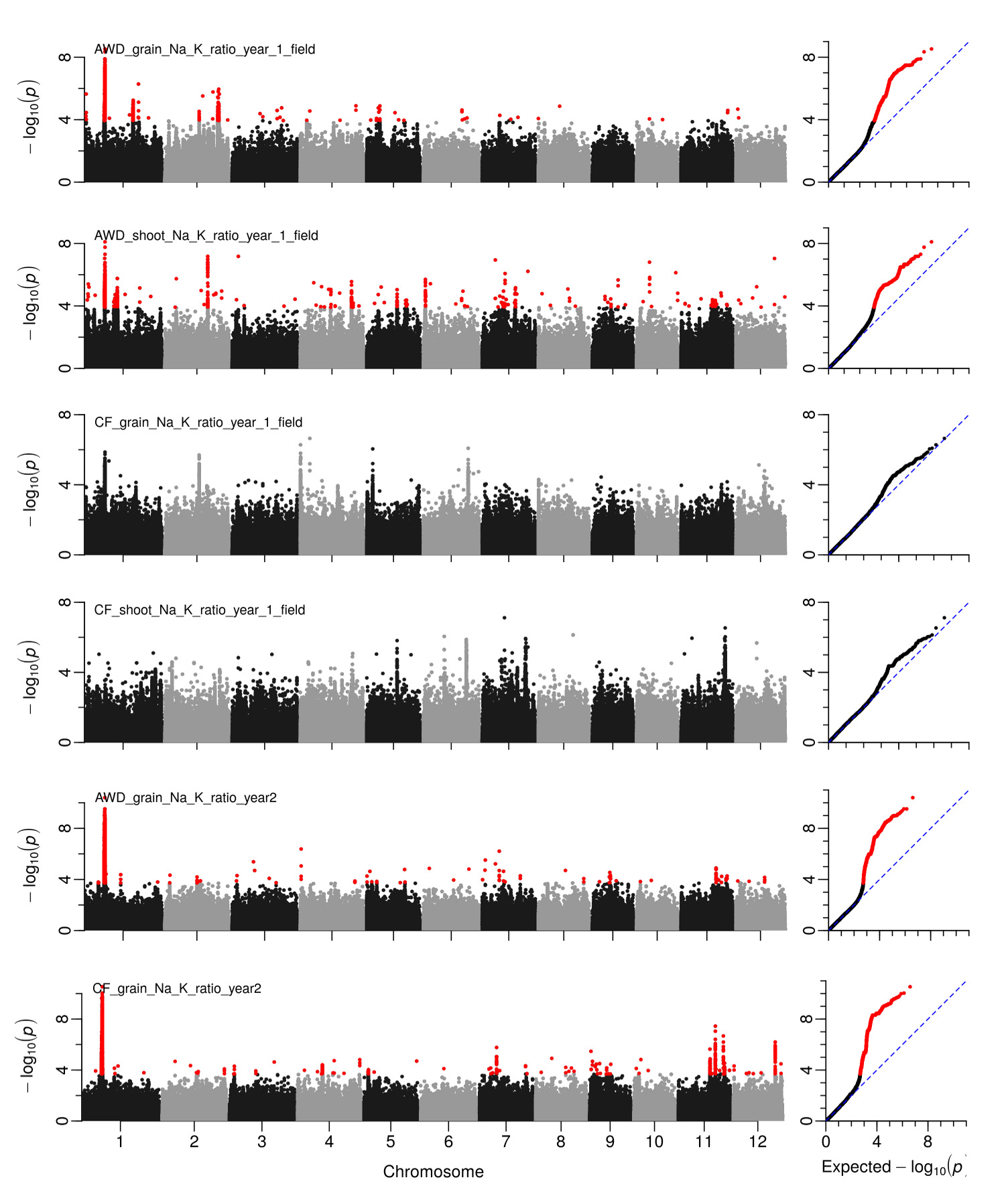

Supplement: Supplementary file 5 — Supplementary Fig. S4 Genome-wide association for Na+/K+ ratio in grains and shoots under CF and AWD. Benjamini-Hochberg adjusted probabilities > 0.1 are highlighted in red dot. The diagonal blue line shown on QQ Plots represents 1:1 agreement between expected probability. (JPG 431 kb) [file 122_2021_3828_MOESM5_ESM.jpg]

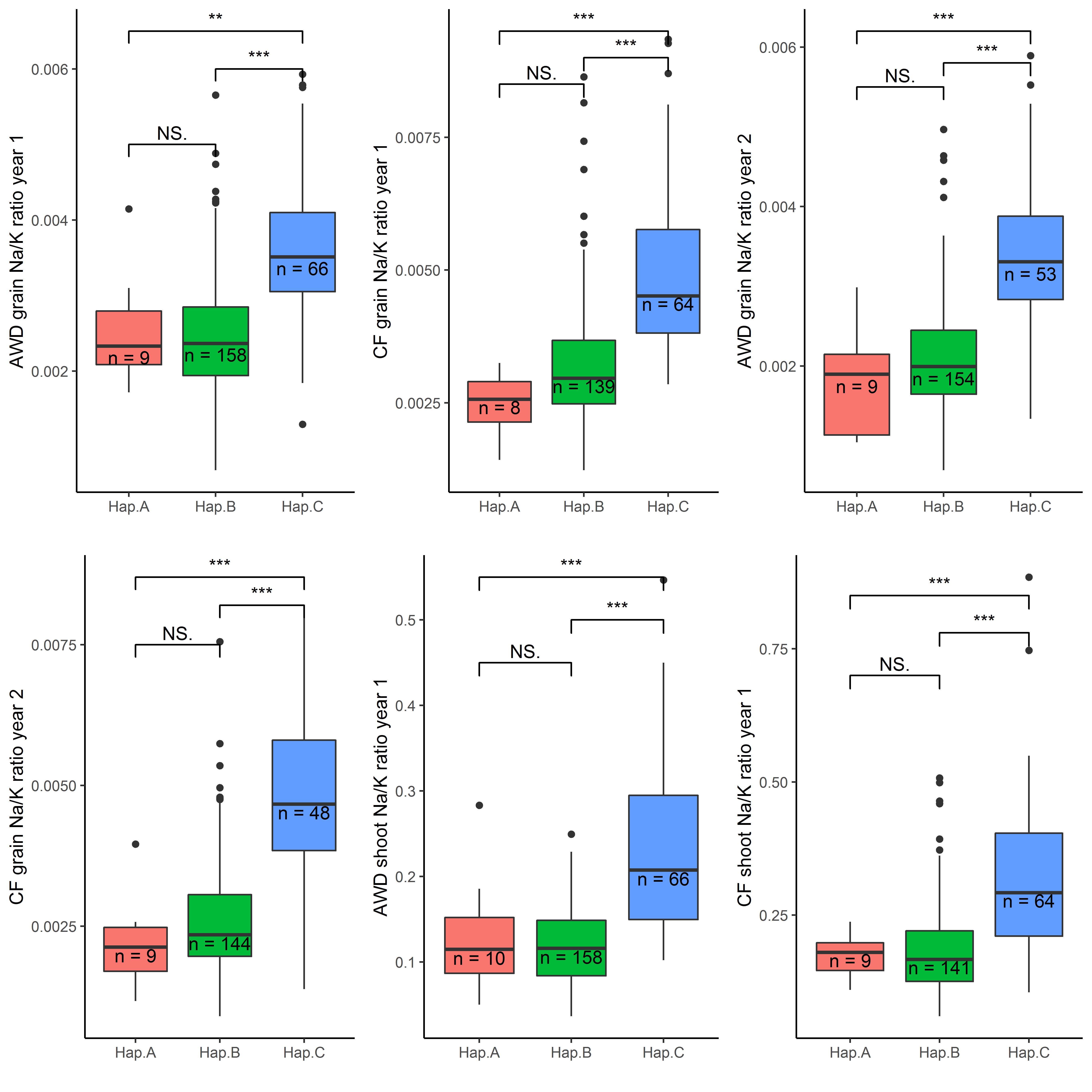

Supplement: Supplementary file 6 — Supplementary Fig. S5. Na+/K+ ratio in grains and shoots for indicated haplotypes of OsHKT1; 5. (JPG 1039 kb) [file 122_2021_3828_MOESM6_ESM.jpg]

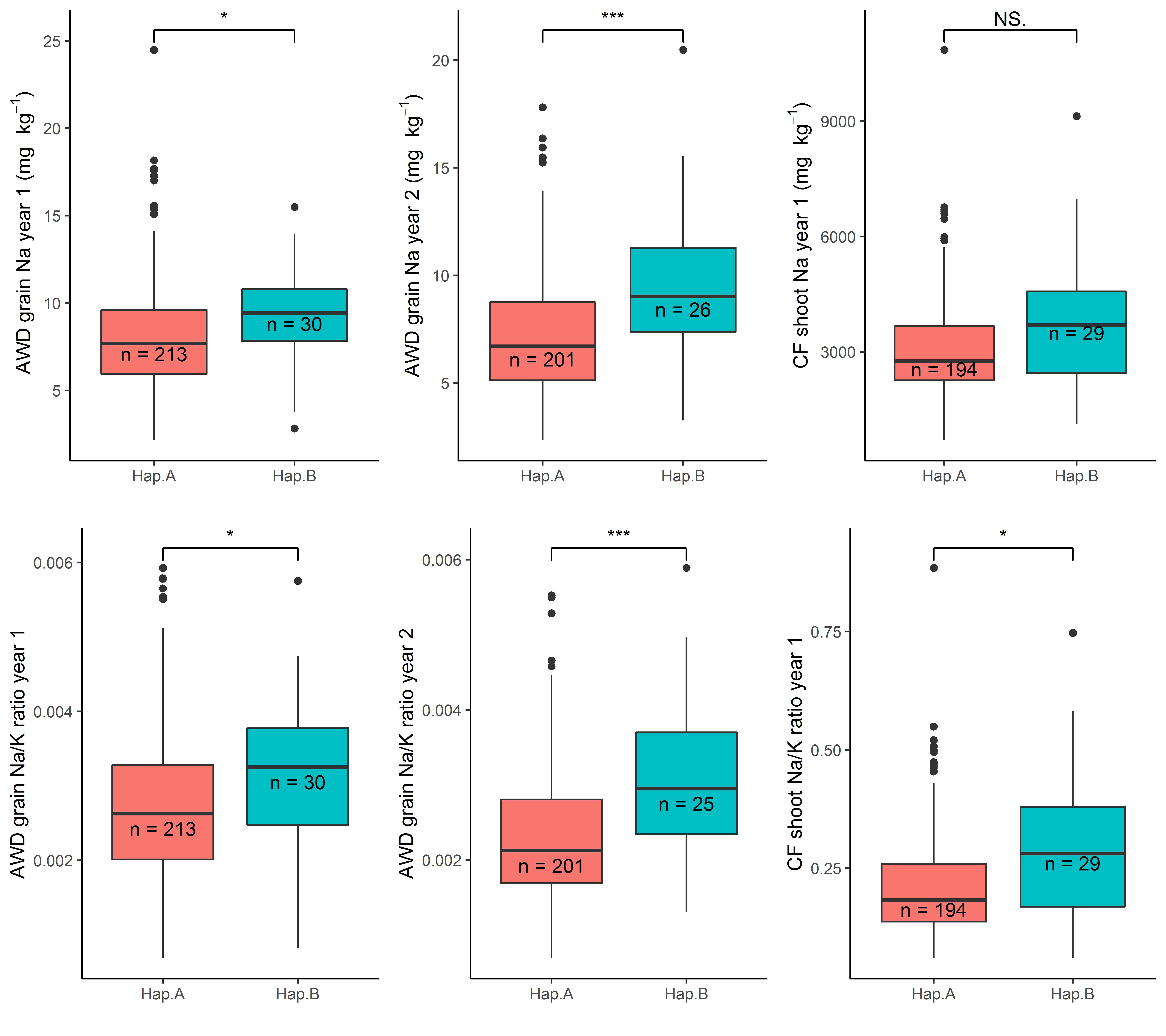

Supplement: Supplementary file 7 — Supplementary Fig. S7. Na+ concentration and Na+/K+ ratio in grains and shoots for indicated haplotypes of OsNHX2. (JPG 862 kb) [file 122_2021_3828_MOESM7_ESM.jpg]
